# Confirmation of interpersonal expectations is intrinsically rewarding

**DOI:** 10.1093/scan/nsab081

**Published:** 2021-07-07

**Authors:** Niv Reggev, Anoushka Chowdhary, Jason P Mitchell

**Affiliations:** Department of Psychology, Harvard University, Cambridge, MA 02138, USA; Department of Psychology, Ben-Gurion University of the Negev, Be’er-Sheva 84105, Israel; Zlotowski Center for Neuroscience, Ben-Gurion University of the Negev, Be’er-Sheva 84105, Israel; Department of Psychology, Harvard University, Cambridge, MA 02138, USA; Department of Psychology, Harvard University, Cambridge, MA 02138, USA

**Keywords:** stereotypes, reward, fMRI, NAcc, value, consistency

## Abstract

People want to interact successfully with other individuals, and they invest significant efforts in attempting to do so. Decades of research have demonstrated that to simplify the dauntingly complex task of interpersonal communication, perceivers predict the responses of individuals in their environment using stereotypes and other sources of prior knowledge. Here, we show that these top-down expectations can also shape the subjective value of expectation-consistent and expectation-violating targets. Specifically, in two neuroimaging experiments (*n *= 58), we observed increased activation in brain regions associated with reward processing—including the nucleus accumbens—when perceivers observed information consistent with their social expectations. In two additional behavioral experiments (*n* = 704), we observed that perceivers were willing to forgo money to encounter an expectation-consistent target and avoid an expectation-violating target. Together, these findings suggest that perceivers value having their social expectations confirmed, much like food or monetary rewards.

People dedicate a substantial portion of their time to interacting with other individuals. When we succeed in doing so, we feel better physically and psychologically (Baumeister and Leary, 1995; Tay *et al.*, 2013; Cacioppo *et al.*, 2014; Matthews and Tye, 2019). At the same time, other people also present one of the most complicated challenges we have to face. Understanding another person involves inferring hidden states based on fragmentary sensory, verbal and visceral cues, each of which conveys only a small amount of information. To simplify the highly demanding challenge of social cognition, perceivers use top-down predictions (e.g. stereotypes) that help make sense of others in a rapid fashion (Freeman and Johnson, 2016; Otten *et al.*, 2017; Tamir and Thornton, 2018; Hutchinson and Barrett, 2019). Perceivers can thus seamlessly interact with their environment while refraining from the effortful construction of elaborative representations for each individual they encounter (Fiske and Neuberg, 1990; Macrae and Bodenhausen, 2000). For example, on her first day of school, a freshman might assume that her female peers may be interested in conversing about shopping. Utilizing these predictions, the freshman could effortlessly engage in spontaneous conversations with her new female peers, potentially facilitating her social bonds.

However, although they often facilitate interpersonal interaction, social predictions also impose a cost on perceivers. Individuals interpret ambiguous information in accordance with their expectations to confirm existing biases (Darley and Gross, 1983). Moreover, across multiple contexts, individuals tend to adhere to predictions they have previously formed and fail to modify them even in the face of contradictory evidence (Hamilton and Sherman, 1996; Gregg *et al.*, 2006; Roese and Sherman, 2007; Wyer, 2010; Dunsmoor *et al.*, 2016). In the person perception domain, when perceivers first learn that someone is ‘intelligent’ and then subsequently discover that he is also ‘envious’, they form a favorably skewed impression of that person; on the other hand, when perceivers learn about these same two character traits in reverse order, they form an unfavorable impression of the target (Asch, 1946; Sullivan, 2019). In the domain of stereotypes about social groups, perceivers typically do not update self-reported or implicitly measured preferences for a social group once formed (Gregg *et al.*, 2006; Roese and Sherman, 2007). Together, such findings provide further evidence that people prefer to have their social predictions confirmed across multiple domains. To date, research has not been able to identify the sources that support the persistence of these initial predictions about other individuals. Here, we integrate insights from social psychology and neuroscience to explore the idea that perceivers prefer to ‘stick’ with their initial predictions because they attribute subjective value to the confirmation of these predictions. That is, we posit that targets who confirm our expectations about them (such as stereotype-consistent targets) will trigger a reward-like response similar to food, sex or chocolate.

Several lines of research already hint at such an effect. Perceivers generally like individuals who conform to expectations more than individuals who violate them (Eagly and Karau, 2002; Phelan and Rudman, 2010; Stern *et al.*, 2015); for example, observers typically prefer female teachers to male teachers, but like male leaders better than their equally competent female peers (Rudman *et al.*, 2012; Moss-Racusin and Johnson, 2016). Similarly, participants express greater trust in targets that fit gender-based predictions (Olszanowski *et al.*, 2018; Stern and Rule, 2018). Moreover, perceivers demonstrate similar effects for emotion-based expectations, regardless of the valence of the emotion (Chanes *et al.*, 2018). Several theorists have suggested that perceivers may gradually develop a habitual hedonic response for targets conforming to normative expectations (Jost and Hunyady, 2003; Berridge, 2012; Huebner, 2016; Theriault *et al.*, 2020). These suggestions dovetail with the well-documented aversive reactions people experience when confronted with violations of predictions and the uncertainty associated with such violations (Festinger, 1957; Roese and Sherman, 2007; Gawronski, 2012; FeldmanHall and Shenhav, 2019; Theriault *et al.*, 2020). In a similar vein, when perceivers attempt to form an impression about an expectation-violating social target, they effortfully process the information (Fiske and Neuberg, 1990) and show enhanced activity in several brain regions, including the dorsomedial prefrontal cortex (Cloutier *et al.*, 2011; Ames and Fiske, 2013; Mende-Siedlecki *et al.*, 2013). Put together, these positive and aversive responses motivate perceivers to seek expectation-consistent information.

In spite of ample evidence for perceivers’ motivation to confirm their social expectations, scholars are still debating the mechanisms supporting the persistence of this motivation. Here we suggest that neural activity can offer a novel insight on this topic. In recent years, scholars have identified the involuntary effects of motivation and expectation in several neural systems, most notably in the mesolimbic dopaminergic system (Kohli *et al.*, 2018). Animal models suggest that midbrain dopaminergic activity signals one’s internal desire to obtain a goal (Berridge, 2012). In humans, the motivation to experience positive effects manifests specifically in midbrain and striatal responses to better- or worse-than-expected information (Sharot *et al.*, 2012; Lefebvre *et al.*, 2017; Charpentier *et al.*, 2018). Likewise, participants expecting a painful stimulus demonstrate increased striatal activity while experiencing pain, compared with participants who do not expect to feel pain (Jepma *et al.*, 2018; Schwarz *et al.*, 2019; for a related effect of negative stigma, see Welborn *et al.*, 2020). Finally, a recent meta-analysis reported that when perceivers agreed with expected group opinions, they demonstrated robust striatal activity compared with times in which they deviated from the group consensus (Wu *et al.*, 2016). These studies suggest that insofar as perceivers hold a motivation to experience a specific event, the striatum responds to events that align with that motivation.

Notably, the involvement of the striatum hints at a potential mechanism driving the effects of expectation and motivation. Researchers repeatedly identify striatal activity, and most prominently activity in its ventral portion, in anticipation and receipt of various types of reward (Schultz, 2000; Hare *et al.*, 2008; Haber and Knutson, 2010). For example, the nucleus accumbens (NAcc), located at the ventral–rostral tip of the striatum, responds both to primary rewards (e.g. food or erotic) and secondary rewards (e.g. money or positive feedback) (Peters and Büchel, 2010; Bartra *et al.*, 2013; Sescousse *et al.*, 2013). The NAcc also responds to social experiences, such as engagement with attractive or smiling faces, prosocial actions or placing one’s trust in peers (Harris and Fiske, 2010; Lin *et al.*, 2012; Hackel *et al.*, 2015; Krosch and Amodio, 2019). As increased striatal responses are often associated with rewards, such a response to events aligning with perceivers’ motivation might indicate that these events are rewarding as well.

Together, these studies suggest that consistency with stereotypes and other forms of interpersonal predictions is intrinsically rewarding. To test this hypothesis, we first measured NAcc activity in response to information consistent or inconsistent with social expectations. Using functional magnetic resonance imaging (fMRI), we scanned participants while they judged target individuals on characteristics that either conformed to or violated interpersonal expectations. As the NAcc is consistently involved in rewarding experiences, activity in this region can serve as a marker of a neural reward response. If perceivers value having their social expectations confirmed, we should observe increased NAcc activity for trials in which targets were associated with characteristics that confirm expectations compared to trials in which targets violate them.

In addition, we assessed whether perceivers actively prefer expectancy-confirming social information by creating experimental situations in which participants could trade money for the chance to view targets with expectation-consistent characteristics. To do so, we relied on a modified version of a ‘pay-per-view’ task, previously used with human and non-human primates, to measure the monetary value associated with expectancy-confirming and expectancy-violating stimuli (Deaner *et al.*, 2005; Tamir and Mitchell, 2012). If perceivers experience expectancy-confirming information as intrinsically more valuable, we expected participants to forgo money to rate individuals associated with expectancy-consistent rather than expectancy-violating information.

Interestingly, social expectations can take multiple forms. For example, stereotypes relate specific attributes to social groups regardless of personal knowledge about group members. Conversely, we can construct detailed individuated expectations about familiar individuals, such as personally familiar others or famous people (Fiske and Neuberg, 1990; Hamilton and Sherman, 1996). Accordingly, we also probed whether predictions from these two distinct sources evoked qualitatively different reward responses. Together, the results of four studies support the hypothesis that perceivers are motivated to reaffirm their interpersonal forecasts, in part because they experience the confirmation of social expectations as a powerful form of subjective reward.

## Results

### Study 1: neural response to stereotype confirmation *vs* violation

**Fig. 1.**
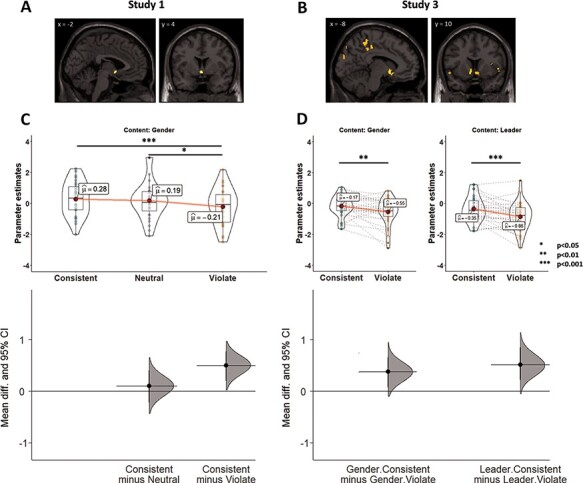
Neural responses associated with the confirmation of expectations about other individuals. Whole-brain random-effects contrasts comparing expectation-consistent > expectation-violating trials revealed activity in the NAcc in (A) Study 1 and (B) Study 3. In addition, we independently defined an ROI in the NAcc using a comprehensive meta-analysis (MNI coordinates: −6, 10, −6; 10, 12, −6). Analysis of parameter estimates in this region confirmed that the bilateral NAcc showed a stronger response during consistent than during violating trials in (C) Study 1 and (D) Study 3. Upper panel: Across all figures, individual dots represent parameter estimates for individual participants. Each figure also visualizes the mean of each condition (as a red dot), the median (solid horizontal line), and the first and third quartiles (boxplot). Lower panel: effect size (the mean difference between respective conditions, indicated by the black circles), the bootstrapped 95% CIs (illustrated by the vertical lines) and the resampled distribution of the effect size given the observed data, indicated by the curve (see Methods section).

In Study 1, we observed greater NAcc activity when participants saw targets associated with gender-stereotype-consistent information than when they saw targets associated with stereotype-violating information. We scanned participants (*n* = 28; here and in subsequent studies, the sample size reported includes only participants who did not fail pre-defined exclusion criteria; see Methods section) while they formed impressions about targets that varied in the degree to which they confirmed gender stereotypes. Gender stereotypes included various characteristics typically associated with men (e.g. ‘emotionally closed’ or ‘CEO of a big company’) or women (e.g. ‘loves children’ or ‘an admired preschool teacher’; all characteristics were piloted in advance, see Supplementary data available online). In each of the 204 trials, participants first read a short description for 1.5 s and then saw the face of a target man or woman (Supplementary Figure S1A). Participants rated how likely the target was to have the presented characteristic (see Supplementary data results and Supplementary Table S1 for behavioral results). We conducted three parallel analyses to examine whether the neural region most associated with reward—namely, the NAcc—was more engaged when the target was associated with stereotype-derived expectations compared to when the target violated them. First, a whole-brain random-effects contrast identified regions that were more active for ‘stereotype-consistent’ > ‘stereotype-violating’ trials (*P* < 0.05, corrected; Supplementary Table S2 for full results). This analysis indicated a significantly greater response in the NAcc when the presented target matched the stereotypical expectation set by the preceding statement than when the target violated that expectation (Figure 1A). Similar results were obtained when we modeled expectation-consistency as a continuous rather than dichotomized predictor (Supplementary Table S2 and Supplementary Materials and Methods for full details).

Second, to confirm that this region overlapped with those responsive to rewards, we independently defined neural regions of interest (ROIs) based on spheres around peak voxels identified in a comprehensive meta-analysis (Bartra *et al.*, 2013). In this independently defined region, consistency with stereotypes resulted in significantly greater activity compared to their violation (one-sided test: *t*_(54)_ = 3.53, *P *= 0.0004, Hedges’s *g* = 0.37 [95% confidence intervals (CIs): 0.1–0.64], Figure 1B). Third, we corroborated this finding by defining ROIs from a task in which participants received monetary rewards based on their performance [the Monetary Incentive Delay (MID) task; see Materials and Methods] (Knutson *et al.*, 2000). This analysis yielded similar results (*t*_(54)_ = 2.87, *P *= 0.0029, *g* = 0.30 [0.03–0.59]). Together, these patterns suggest that seeing a person associated with a stereotypical characteristic triggers activation in the very same region that responds to primary and secondary reinforcers, highlighting the intrinsic value of stereotype confirmation.

**Fig. 2.**
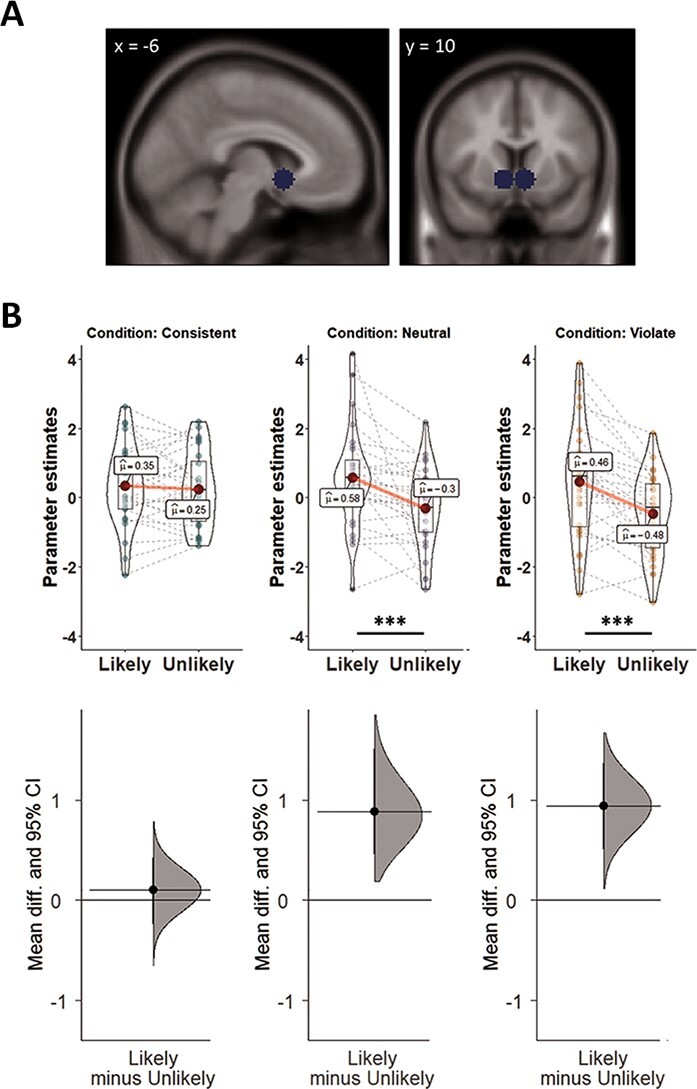
The effect of behavioral ratings of trials on neural responses. In each trial, participants indicated whether they thought the presented target was likely or unlikely to be associated with the presented statement. (A) The independently defined ROIs in the NAcc. (B) We observed a significant interaction in the independently defined NAcc in Study 1: Participants’ ratings modulated the neural response only for stereotype-neutral and stereotype-violating targets, suggesting that stereotype-confirming targets are involuntarily rewarding.

Additionally, to investigate whether this NAcc activation is limited to stereotype-derived expectations, we included a third type of statements in our study: stereotype-neutral statements (e.g. ‘drinks coffee every morning’). Interestingly, we found that the overall NAcc response to targets associated with stereotype-neutral information was higher than the response to stereotype-violating information and not different from stereotype-consistent information (Figure 1C; Bonferroni corrected comparisons: neutral *vs* violating: Meta-analysis ROIs: *t*_(54)_ = 2.83, *P* = 0.0195, *g* = 0.29 [0.09–0.51]; MID ROIs: *t*_(54)_ = 2.38, *P* = 0.062, *g* = 0.25 [0.04–0.47]; neutral *vs* consistent: Meta-analysis ROIs: *t*_(54)_ = 0.7, *P* = 0.76, *g* = 0.07 [−0.19–0.34]; MID ROIs: *t*_(54)_ = 0.49, *P* = 0.63, *g* = 0.05 [−0.18–0.28]). At first blush, this finding suggests that NAcc activation was modulated only by stereotype-inconsistent information, not that it was especially driven by stereotype-consistent information which, on average, did not differ from stereotype-neutral trials. However, additional analyses belie this interpretation. On each trial, participants rated the likelihood that the target could be described by the accompanying characteristic (e.g. ‘enjoys drinking coffee in the morning’). Importantly, the NAcc was activated during stereotype-neutral trials only when participants endorsed those characteristics as descriptive of the target and not when they rejected its applicability to a target (meta-analysis ROIs: *F*_(1,76.18)_ = 15.67, *P *= 0.0002; *η_p_^2^* = 0.17 [0.06, 0.29]; MID ROIs: *F*_(1,76.5)_ = 12.66, *P *= 0.0006; *η_p_^2^* = 0.14 [0.04, 0.26]). In contrast, the preferential NAcc activation observed for stereotype-consistent trials was unaffected by participants’ likelihood ratings (interaction between response and condition: meta-analysis ROIs: *F*_(1.77,47.68)_ = 5.4, *P *= 0.01, *η_p_^2^* = 0.17 [0.02, 0.3]; MID ROIs: *F*_(1.87,50.4)_ = 4.01, *P *= 0.03, *η_p_^2^* = 0.13 [0.01, 0.26]; see Figure 2. Stereotype-consistent trials: meta-analysis ROIs: *F*_(1,76.18)_ = 0.2, *P *> 0.5; *η_p_^2^* = 0.003 [0, 0.05]; MID ROIs: *F*_(1,76.5)_ = 0.63, *P *> 0.5; *η_p_^2^* = 0.005 [0, 0.06]). Together, these data points suggest that NAcc is activated specifically when one’s expectations are confirmed, regardless of whether those expectations derive from culturally salient stereotypes or from more personal and idiosyncratic sources. Finally, as for stereotype-neutral trials, stereotype-violating trials (e.g. a man who ‘enjoys shopping for shoes’) were associated with greater NAcc activation when participants endorsed the likelihood of such characteristics (meta-analysis ROIs: *F*_(1,76.18)_ = 17.84, *P *= 0.0001; *η_p_^2^* = 0.19 [0.07, 0.31]; MID ROIs: *F*_(1,76.5)_ = 15.76, *P *= 0.0002; *η_p_^2^* = 0.17 [0.06, 0.29]).

### Study 2: the monetary value of stereotype confirmation

Although activation of the NAcc often reflects the presence of rewarding stimuli (Bhanji and Delgado, 2014), this region can also respond to non-value-related processes, including information coding or salience effects (O’Doherty, 2014). To complement our initial neural results, in the preregistered Study 2 we examined a behavioral measure of the value associated with rating expectancy-confirming targets. Specifically, we tested how much money participants were willing to forgo to view stereotype-consistent instead of stereotype-violating targets.

Participants on Amazon Mechanical Turk (*n* values* *= 174 and 169 in Study 2a and 2b, respectively) made a series of choices to rate one of two target types: a stereotype-consistent target (e.g. a man who enjoys riding motorcycles) or a stereotype-violating target (e.g. a man who enjoys shopping for shoes), designated as ‘typical’ and ‘atypical’, respectively. After each choice, participants saw a target accompanied by a statement and rated the likelihood that the target would be associated with the statement on a 0–100 scale. Participants had up to 5 s for each phase of the task (Supplementary Figure S2). To avoid potentially different responses to male and female targets, Study 2a included only male faces and Study 2b included only female faces. On each of the 25 trials, small monetary payoffs ($0.03–$0.09 in increments of 2 cents) were associated with each target choice. Participants received a subset of these payoffs as a monetary bonus for the task. Payoff amounts for each target choice varied across trials (and were occasionally equal), as did the location of the option for which participants received the larger amount. If consistency with stereotypes is intrinsically rewarding, participants should be willing to forgo money—to choose the lower-paying option—to see stereotype-consistent individuals. On the other hand, a participant seeking to maximize monetary payoff should consistently select the higher paying option regardless of the stereotypicality of the information that follows.

**Fig. 3.**
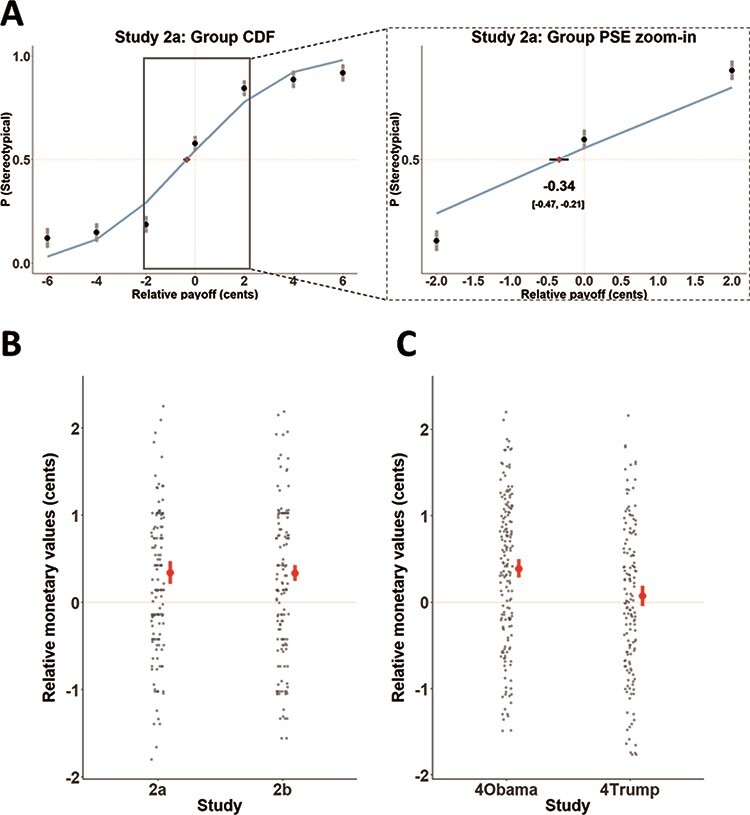
The monetary values of consistency with stereotype and person-specific expectations. (A) Visualization of the cumulative distribution function we used to calculate the PSE, illustrated by group data from Study 2a. The x-axis represents the difference between the monetary values associated with the two target types presented in each trial. Each dot indicates the proportion of trials in which participants chose to rate a stereotype-confirming over a stereotype-violating target. The PSE was calculated as the point at which a cumulative normal distribution function, fit to these responses, passes 50%. This point represents the relative monetary value associated with one target type over the other. Negative values indicate that participants preferred to incur a relative monetary loss to rate a confirming target. Error bars depict 95% CIs. (B) Distribution of individual PSE values for Study 2. Rating stereotype-consistent targets in Studies 2a and 2b was associated with significantly higher subjective value than rating stereotype-violating targets. Each gray dot depicts PSE for a specific participant. Red dots indicate the sample mean. Error bars depict 95% CIs. (C) Distribution of individual PSE values for Study 4. Rating Obama-consistent trials was associated with significantly higher subjective value than rating Obama-violating trials. However, rating Trump-consistent trials did not significantly differ from rating Trump-violating trials.

We modeled the relative value of each target type by calculating the point of subjective equivalence (PSE) between the two options. This value was derived by fitting a cumulative normal distribution curve to participants’ choices (Figure 3A) and finding the monetary value at which participants effectively chose arbitrarily between the two target types (Moscatelli *et al.*, 2012). Thus, the PSE represents the relative monetary value of one target type over another.

As predicted, participants demonstrated a significant preference for seeing stereotype-consistent targets over stereotype-violating targets. When the two trial types shared the same payoff amounts, participants chose the stereotypical targets 58% and 57% of the time for male and female targets, respectively (significantly more than chance, as indicated in a generalized mixed model analysis by an odds ratio of 1.4, *Z* = 3.11, *P* = 0.0019 and an odds ratio of 1.41, *Z* = 2.91, *P* = 0.00369 for the zero-centered intercept in Study 2a and 2b, respectively). Moreover, the calculated PSE indicated that participants forewent an average of 0.34 and 0.335 cents per trial to rate a stereotype-consistent over a stereotype-violating target in Study 2a and 2b, respectively (95% CI: 0.21–0.47, *t*_(173)_ = 5.17, *P* < 0.0001, Cohen’s *d* = 0.39 [0.24–0.55] and CI: 0.25–0.43, *t*_(168)_ = 7.3, *P* < 0.0001, *d* = 0.56 [0.4–0.72] for the two studies, respectively; Figure 3B; Supplementary Table S6 for choice distribution across all trials). This PSE resulted in an average loss of 10% of potential earnings, as participants chose lower monetary amounts to view stereotype-consistent targets. Just as non-human primates prefer to view dominant groupmates over receiving juice (Deaner *et al.*, 2005) and students are willing to forgo money to talk about themselves (Tamir and Mitchell, 2012) or to view attractive members of the opposite sex (Hayden *et al.*, 2007), our participants gave up money to view information that was in line with their stereotypical expectations.

### Study 3: neural response to interpersonal expectations

Together, Studies 1 and 2 suggest that perceivers experience consistency with social expectations as intrinsically rewarding. However, we designed these studies primarily to test the effects of a specific type of social expectations—gender-based stereotypes. Although stereotypes are a significant source of interpersonal expectations, perceivers routinely use additional, idiosyncratic sources of information, especially for individuals with whom they are highly familiar. Does the reward value of expectancy-consistent information extend to person-specific predictions?

To examine this question, Study 3 assessed the responses of the neural reward system to the consistency with and violation of expectations regarding two highly familiar targets, the current and previous presidents of the USA at the time of the study: Donald Trump and Barack Obama, respectively. To facilitate comparison to Study 1, the preregistered Study 3 also included stereotype-derived expectations about unfamiliar targets. As in Study 1, participants (*n* = 30) rated how likely each of 240 specific statements described a specific man or woman or described Donald Trump or Barack Obama. We presented stereotype-related and person-specific statements in blocks of 15 trials per content domain for a total of 60 trials per condition (consistent/violating targets in the stereotype/person-specific domain). Person-specific statements included various characteristics typically associated with one—but not the other—leader (e.g. ‘Supports a wall along the borders’ *vs* ‘Acts to support women’s rights’). Participants first read a short statement for 1.5 s and then saw the face of a target (a man, a woman, Trump, or Obama; Supplementary Figure S1C). Next, participants rated how likely the statement was to describe the target (Supplementary Table S3 and Supplementary Results for full details).

We conducted two parallel analyses to examine whether the NAcc was more engaged when presented with information that was consistent with expectations than with information that violated them. First, a whole-brain random-effects contrast identified regions that were more active for ‘expectation-consistent’ > ‘expectation-violating’ trials (*P *< 0.05, corrected; Supplementary Table S4 for full results). This analysis indicated significantly greater response in several regions, including the NAcc, when the target was consistent with the expectation set by the preceding statement than when the target violated that expectation (Figure 1B).

In a second analysis, we defined the NAcc via two independent procedures, first as bilateral spheres around peak voxels identified in a meta-analysis, and then by examining participants’ neural responses during the MID task. Across both procedures the NAcc demonstrated a robust difference in activity between expectation-consistent and expectation-violating targets (Figure 1D). However, we did not observe any difference between stereotypes and person-specific trials in any of the analyses. Specifically, a 2 × 2 repeated measures analysis of variance (ANOVA) over activity in bilateral NAcc revealed a main effect of expectation-consistency, with higher activation associated with expectation-consistent compared to expectation-violation (meta-analysis ROIs: *F*_(1,29)_ = 21.89, *P* < 0.0001, *η^2^_p_* = 0.43 [0.19–0.58]; MID ROIs: *F*_(1,29)_ = 11.2, *P* = 0.002, *η^2^_p_* = 0.28 [0.07–0.46]). Activation did not significantly differ between stereotype and person-specific content (meta-analysis ROIs: *F*_(1,29)_ = 3.64, *P* = 0.07, *η^2^_p_* = 0.11 [0–0.29]; MID ROIs: *F*_(1,29)_ = 1.41, *P* = 0.24, *η^2^_p_* = 0.05 [0–0.2]) and no interaction was observed between the factors (meta-analysis ROIs: *F*_(1,29)_ = 0.31, *P* = 0.58, *η^2^_p_* = 0.01 [0–0.13]; MID ROIs: *F*_(1,29)_ < 0.01, *P* = 0.99, *η^2^_p_* < 0.0001). Accordingly, expectation-consistency yielded more neural activity for each of the two content domains when examined separately (meta-analysis ROIs: *F*_(1,54.8)_ = 5.96, *P* = 0.0179, *η^2^_p_* = 0.1 [0.01–0.23] and *F*_(1,54.8)_ = 11.03, *P* = 0.0016, *η^2^_p_* = 0.17 [0.04–0.31] for gender stereotypes and person-specific expectations, respectively; MID ROIs: *F*_(1,55.47)_ = 4.36, *P* = 0.04, *η^2^_p_* = 0.07 [0.001–0.2] and *F*_(1,55.47)_ = 4.45, *P* = 0.04, *η^2^_p_* = 0.07 [0.002–0.2], respectively). Thus, consistency with social expectations triggered more activation in the NAcc than violation of such expectations, regardless of whether source of the expectation was general social knowledge (stereotypes) or person-specific knowledge.

Finally, similar to Study 1, variability in behavioral responses in Study 3 enabled us to test the effects of subjective endorsement of characteristics for stereotype-based characteristics (but not for person-specific-expectations; see Supplementary Results). As in Study 1, NAcc activity was not modulated by subjective endorsement of characteristics for stereotype-consistent targets (simple effects analyses: meta-analysis ROIs: *F*_(1,58)_ = 3.99, *P* = 0.0503; *η_p_^2^* = 0.06 [0, 0.18]; MID ROIs: *F(*_1,56.5)_ = 3.20, *P* = 0.08; *η_p_^2^* = 0.05 [0, 0.17]). However, we did not observe an interaction between response and condition (meta-analysis ROIs: *F*_(1,29)_ = 0.08, *P* > 0.5, *η_p_^2^* = 0.003 [0, 0.09]; MID ROIs: *F*_(1,29)_ = 0.40, *P* > 0.5, *η_p_^2^* = 0.01 [0, 0.14]). In other words, unlike in Study 1, NAcc activation was insensitive to the degree to which a participant judged information to apply to a target, regardless of the stereotypicality of the target. Together, these results suggest that consistency with expectations about other targets is valuable for the two most dominant sources of interpersonal expectations—group-based stereotypes and person-specific knowledge.

### Study 4: the monetary value of person-specific expectation confirmation

In Study 2, we observed that perceivers are willing to forgo money to view stereotype-consistent (rather than stereotype-violating) information. To examine whether this behavioral effect extends to expectations about specific individuals, Study 4 replicated the procedure from Study 2 using familiar individuals (Obama and Trump). On each of 32 trials, participants on Prolific Academic (*n* values = 189 and 172 in Study 4a and 4b, respectively) first chose between seeing either an expectation-consistent or an expectation-violating target and then rated the target. Study 4a included only Obama as the target of statements, and Study 4b included only Trump. After choosing the type of content they would like to see, participants rated the likelihood that a specific statement would be associated with the target on a 0–100 scale. Payoff amounts for each choice varied across trials (and were occasionally equal), as did the option for which participants received the larger amount. As in Study 2, we quantified the subjective monetary value of each option by calculating the PSE between the two display types by fitting a cumulative normal distribution curve to participants’ choices and finding the monetary value at which participants were indifferent to the two options. If participants experience consistency with expectation as equally rewarding regardless of the target of expectations, then they should choose to forgo money to rate statements consistent with Obama and Trump in Studies 4a and 4b, respectively.

The results from Study 4a indicate that participants preferred to see expectation-consistent statements of Barack Obama. When the payoff amounts were equal for confirming and violating statements, participants chose the consistent statements 60% of the time (significantly more than chance, as indicated in a generalized mixed model analysis by an odds ratio of 1.42, *Z* = 4.74, *P* < 0.0001 for the zero-centered intercept in Study 4a). Moreover, the calculated PSE indicated that, on average, participants gave up 0.39 cents per trial to rate an expectancy-consistent statement over an expectancy-violating statement about Obama (95% CI: 0.28–0.49, *t*_(188)_ = 7.21, *P *< 0.0001, *d* = 0.52 [0.37–0.68]; Figure 3C). However, the same was not true for Trump-related statements in Study 4b. At equal payoff amounts, participants had no preference between expectancy-consistent and expectation-violating statements (choosing the consistent option 49% of the time; odds ratio of 1.01, *Z* = 0.13, *P* = 0.9), and the calculated PSE was not different from zero (0.07 cents; [−0.05–0.19], *t*_(173)_ = 1.17, *P* = 0.12, *d* = 0.09 [−0.06–0.24]; Figure 3C). The difference between the studies was significant (*t*_(328.29)_ = 2.28, *P* = 0.023, *d* = 0.25 [0.03–0.46]). However, this finding should be interpreted with caution, as our sample was demographically skewed toward liberals, potentially diluting the effect of consistency with expectations from Trump with additional factors (Supplementary Figure S3). Together, these findings suggest that the rewarding effect of expectation-consistency is not limited to stereotypes but also applies to knowledge about specific individuals. Notably, however, not all sources of knowledge equally contribute to the reward value; people were unwilling to forgo monetary amounts to rate content consistent with their expectations about Donald Trump (see Supplementary Results, Supplementary Figure S4 and Supplementary Table S5 for potentially related findings in Study 3).

## Discussion

The human preference for consistent and predictable social interactions has long been acknowledged as a core motivational component driving everyday behavior (Festinger, 1957; Gawronski, 2012). To predict the behavior of others, perceivers regularly employ biased strategies to collect and interpret information that corresponds to their expectations (Lord *et al.*, 1979; Johnston and Macrae, 1994; Frimer *et al.*, 2017; Falben *et al.*, 2019; Oyserman and Yan, 2019). Here we provide evidence to suggest that humans associate expectation-consistent information with intrinsic value, much like other forms of reward such as food or money. Our findings suggest that this reward value is generated regardless of the source of the social expectation. Participants were willing to forgo money to rate an expectation-consistent target rather than its expectancy-violating counterpart. Moreover, doing so was associated with increased activity in a brain region in the neural reward circuitry, regardless of whether the target was consistent with gender stereotypes or knowledge about US presidents. Put simply: people find it rewarding to have their (stereotypical or idiosyncratic) social expectations confirmed.

This line of research coincides with emerging theories that highlight the instrumental value of behaviors and perceptions that fall in line with our expectations. In an early example of this value, Allport (1954) described a mental process in which ‘A Scotsman who is penurious delights us because he vindicates our prejudgment’ (p. 22). Some recent theories suggest that, because most of our social expectations are anchored in our social environment, repeated interaction with expectancy-confirming information leads to continuous reinforcement of our expectations (Huebner, 2016; Oyserman and Yan, 2019; Peters, 2020). Once established, these expectations induce motivations and cognitive representations that persist even in the face of disconfirmation (Berridge, 2012; Hughes and Zaki, 2015; Uusberg *et al.*, 2019; Yon *et al.*, 2019). One theory further suggests that the metabolic costs associated with the violation of expectations increase the desirability of expectation-consistent behavior from an evolutionary standpoint (Theriault *et al.*, 2020). In line with these theories, the current studies demonstrate that information consistent with social expectations is indeed associated with subjective value.

The current findings provide a neural extension to prominent accounts of implicit (i.e. involuntary) stereotyping and prejudice (Tibboel *et al.*, 2015; Greenwald and Lai, 2020). Group-based stereotypes typically draw on categorical distinctions to facilitate easier decision-making by enabling faster and more efficient processing of stereotype-confirming information (Roese and Sherman, 2007). Stereotypes are also more familiar, thus allowing perceivers to process such information fluently (e.g. Smith *et al.*, 2006). Downstream, perceivers evaluate expectation-confirming individuals more positively, allocate more economic resources to them, and judge them as more hirable (Phelan and Rudman, 2010; Stern *et al.*, 2015; Stern and Rule, 2018). Complementarily, violations of social expectations pose a threat to individuals and social structures alike, which, in turn, often try to eliminate the threat and reinforce the original expectation (Morgenroth and Ryan, 2020). Our results provide a candidate mechanism for these effects, whereby the preference of expectation-consistent information translates into a subjective value that shapes how we evaluate specific individuals (cf., Amodio and Devine, 2006). Although our findings are mute with respect to the specific mechanism driving the rewarding effect (e.g. a motivational goal to confirm expectations or processing fluency), our results hint at when perceivers can assign value to expectation-violating information. In our task, behavior asymmetrically affected the neural response in the NAcc. Whereas stereotype-consistent targets always evoked the same level of neural activity regardless of participants’ responses, in Study 1 stereotype-violating targets elicited enhanced NAcc activity only if perceivers judged them as likely to be associated with the expectancy. This pattern suggests that participants can assign value to stereotype-violating targets, perhaps depending on the believability of the counter-stereotypical judgment.

The asymmetric effect of behavior on the neural response in the NAcc also suggests that our findings do not result only from participants’ desire to be correct in their predictions. Previous studies used objectively measurable performance to demonstrate increased ventral striatum activity when participants provided correct responses, either with external feedback (Ullsperger and von Cramon, 2003; Tricomi and Fiez, 2008) or in its absence (Satterthwaite *et al.*, 2012; Ruissen *et al.*, 2018). If the correspondence between the targets and predictions would have been the sole process driving the effects, then behavioral ratings of targets would not have interacted with types of prediction to modulate NAcc activity. Nonetheless, future studies should experimentally control for this alternative interpretation by, for example, including an explicit prediction phase before presenting a target and yoking the identity of the target to participants’ predictions.

The expectation-consistency account of ventral striatum activity we put forward complements earlier hypotheses that activity in the mesolimbic circuit reflects prediction errors (Daw *et al.*, 2011). Prediction errors refer to discrepancies between the actual and expected outcomes, typically in the context of learning (Niv and Schoenbaum, 2008). Numerous studies have found that the ventral striatum increases its activity as the gap between the expected and the received outcome grows (e.g. Glimcher, 2011; Ballard *et al.*, 2018). However, the present results diverge from this prediction error pattern. We observed increased striatal activity when an outcome—the target face—was ‘not’ different from the expected outcome; a prediction error account would suggest that such an activity should accompany unexpected information instead. Notably, unlike the vast majority of studies exploring the prediction error account, the paradigms we used in the current investigation did not involve learning. Participants saw each target only once during the entire study and formed their expectations based solely on previously established knowledge. Furthermore, our paradigm did not involve any explicit feedback. Therefore, participants had no objective external verification of their predictions, nor did we directly measure their predictions. Future studies could formally test whether striatal activity in response to expectancy-consistent information also emerges when participants experience feedback and learn new information.

The current set of studies tested the value of expectancy consistency through relatively innocuous stereotypes and associations rather than overtly positive or negative bits of information (with one potential exception of Trump-related statements for liberal participants; see study design section). Including only neutral or mildly valenced statements allowed us to test the direct effects of expectation confirmation with little influence from potentially competing motivations, such as social desirability. Therefore, we cannot determine whether perceivers will continue to value stereotype-confirming information even when they are motivated to suppress them. Our paradigms provided only indirect evidence on this question. In Study 3, our sample consisted of liberal participants (average rating of 3.37 on a 1–9 scale, ‘1’ denoting extremely liberal and ‘9’ denoting extremely conservative) for whom information consistent with Donald Trump might be aversive. However, these participants demonstrated comparable activation in the neural reward circuitry in response to statements about Donald Trump and Barack Obama, implying that seeing expectation-confirming information is rewarding regardless of the valence of these statements. In Study 4, conversely, participants (mean difference of 64 points on a 0–100 scale in favor of liking Barack Obama; see Supplementary Results) did not choose to incur a cost to see Trump-consistent information, suggesting that additional motivations affected their behavior. These first steps warrant further research to characterize the precise mechanisms contributing to the subjective value perceivers attribute to confirmation of expectation and the subsequent behaviors associated with this value.

An additional and important future route of investigation should explore the generalizability of our findings to non-social contexts. Here we focused on expectations concerning social targets—unfamiliar men and women as well as familiar targets. We cannot ascertain whether similar effects would emerge for non-social expectations, such as expectations about inanimate objects or the weather. However, given some accounts suggesting that social information processing has a preferred status in the primate brain (Adolphs, 2009; Atzil *et al.*, 2018; Lockwood *et al.*, 2020), we might expect that (i) the subjective value attributed to consistency should be greater for the kinds of social expectations studied here and (ii) confirmation of ‘social’ expectations would lead to responses similar to reward-like responses for primary reinforcers like food.

Altogether, these findings join a growing body of literature that characterizes how our prior beliefs modulate information processing to fortify a world view and protect the established expectations (Golman *et al.*, 2017; Charpentier *et al.*, 2018; Jepma *et al.*, 2018; Gershman, 2019; Yon *et al.*, 2019). We suggest that the subjective value imbued upon targets who conform to societal expectations may serve to sustain multiple stereotype- and expectation-induced biases. To mitigate the negative implications associated with these expectations, society will need to acknowledge the subjective value associated with their confirmation.

## Materials and methods

### Study design

#### Materials.

To create expectation-setting statements, we generated a list of verbal statements that described relevant individual preferences, traits, behaviors or professions. Studies 1 and 2 included 136 gender-related and 68 gender-neutral statements (see Supplementary Materials and Methods). We verified the stereotypicality of these statements in a pilot study (*n* = 78) in which participants from the local community indicated how typical the characteristic was for a specific gender on a visual scale of 0 (‘very untypical’) to 100 (‘very typical’; the scale had no other tick marks). Each participant was randomly assigned to rate each statement either for men or for women. Participants were instructed to base their ratings on how they thought the average person would respond. This verification procedure was successful; men were associated with men-stereotypic statements more than women (mean difference: 25.7), and women were associated with women-stereotypic statements more than men (mean difference: 25.1). Overall, the statements contained 2–9 words (mean: 4.69, s.d.: 1.44; no difference between experimental conditions, *P* > 0.2) and 9–45 characters (mean: 26.68, s.d.: 7.69; *P* > 0.18). Studies 3 and 4 further included 120 person-specific statements pertaining to Barack Obama and Donald Trump. A total of 243 participants from Amazon Mechanical Turk rated a sample of 60 of these statements, randomly determined per participant. On each trial, participants indicated how typical the presented characteristic was for the two targets (a separate scale for each target; the two scales were presented simultaneously with a randomly determined order). Obama was associated with Obama-related statements more than Trump (mean difference: 56.1), and Trump was associated with Trump-related statements more than Obama (mean difference: 54.5). Overall, the statements contained 2–9 words (mean: 4.65, s.d.: 1.52; no difference between experimental conditions, *P* > 0.5) and 11–45 characters (mean: 28.33, s.d.: 8.43; *P* > 0.5).

#### Studies 1 and 3.

Twenty-eight individuals participated in Study 1 and 30 individuals participated in Study 3. Additional participants were excluded due to excessive motion, technical issues or lack of response to more than 20% of trials (3 and 6 participants from Studies 1 and 3, respectively). All participants provided informed consent in a manner approved by the Committee on the Use of Human Subjects in Research at Harvard University. Study 3 was preregistered (https://osf.io/h9c6x/). See Supplementary data for demographic information. The current sample size allowed a power of 0.8 to detect a medium effect size (Cohen’s *d* = 0.5) in the planned one-tailed contrast between expectation-consistent and expectation-violating targets at the ROI. In both studies, participants formed impressions about target individuals. On each trial, participants first saw a statement for 1.5 s. The statements in Study 1 described a stereotypically neutral, stereotypically male or stereotypically female characteristic; statements in Study 3 described a characteristic that was either stereotypically male or female or closely associated with Barack Obama or Donald Trump (see https://osf.io/tgja3/ for open materials). Next, participants saw the statement with a face of a man or a woman (in Study 1; Study 3 also included face images of the relevant leaders). The statement–face pair appeared on screen for four additional seconds (3.5 s in Study 3) for a total of 5.5 s (5 s) per trial. Each trial ended with a 0.5 s fixation crosshair. Participants used their left hand to indicate how likely the presented target was to be described by the specific characteristic using a 4-point scale (1—‘very unlikely’; 4—‘very likely’). Participants responded while the pair appeared on the screen. Trials in both studies were separated by variable intertrial intervals of 0–9 s (Dale, 1999) optimized for our contrast of interest (see Supplementary Materials and Methods for details).

#### Studies 2 and 4.

A total of 343 participants were included in Study 2 (174 in Study 2a) and 361 in Study 4 (189 in Study 4a). Additional participants were excluded by criteria set in the preregistered protocols for each study (see Supplementary Materials and Methods for details; see also https://aspredicted.org/k4yc2.pdf (Study 2a), https://aspredicted.org/45t3z.pdf (Study 2b) and https://aspredicted.org/s8hs6.pdf (Studies 4a and 4b)). The sample size was set to allow sufficient power (0.8) to detect a small effect size (Cohen’s *d* = 0.2) in a one-sample *t*-test for each study. Informed consent was obtained from all participants in a manner approved by the Committee on the Use of Human Subjects at Harvard University.

### Statistical analysis

#### Studies 1 and 3.

To localize brain regions associated with the processing of rewarding stimuli, we defined 8-mm spheres around peak coordinates drawn from a comprehensive meta-analysis (Bartra *et al.*, 2013). To functionally identify these brain regions, participants in both studies completed a MID task (Knutson *et al.*, 2000) immediately after the impression formation task (Supplementary Figure S1B and Supplementary Materials and Methods for details). This task allows the identification of monetary-reward-sensitive ROIs by comparing trials in which participants won money to trials in which participants could not earn any reward. We extracted and averaged parameter estimates across voxels in each ROI per condition of interest and analyzed them using within-participant ANOVAs as implemented by afex package (Singmann *et al.*, 2018) for R, version 0.22-1. We plotted the results using the package ggstatsplot (Patil, 2021), version 0.2.0. Additionally, we plotted the mean effect size and the bootstrapped 95% CIs using the package dabestr (Ho *et al.*, 2019), version 0.2.2.

We collected neuroimaging data with a 3T Siemens Prisma scanner system (Siemens Medical Systems, Erlangen, Germany). First, we acquired high-resolution anatomical images using a T1-weighted 3D Magnetization Prepared Rapid Acquisition Gradient Echo (MPRAGE) sequence. Next, whole-brain functional images were collected using a simultaneous multi-slice (multiband) T2*-weighted gradient echo sequence (Time of Repetition (TR) = 2000 ms, Echo Time (TE) = 30 ms, voxel size = 2 × 2 × 2 mm^3^, 75 slices auto-aligned to −25° of the anterior commissure (AC) - posterior commissure (PC) line). Participants completed four impression formation task runs consisting of 229 volumes each (245 volumes in Study 3). Finally, participants completed the MID task in a single run consisting of 110 volumes using identical parameters to those mentioned above. We used SPM12 version 6225 (Wellcome Department of Cognitive Neurology, London, UK) to process and analyze the fMRI data. Data were corrected for differences in acquisition time between slices, corrected for inhomogeneities in the magnetic field using fieldmap (Cusack and Papadakis, 2002), realigned to the first image to correct for head movement, unwarped to account for residual movement-related variance, and co-registered with each participant’s anatomical data. Functional data were then transformed into a standard anatomical space (2-mm isotropic voxels) based on the ICBM152 brain template (Montreal Neurological Institute, MNI). Normalized data were then spatially smoothed (6 mm full-width at half-maximum) using a Gaussian Kernel (see Supplementary Materials and Methods for full details of scanning and analysis procedures). We analyzed preprocessed data using a general linear model in which we modeled trials as boxcar functions with an onset at face presentation (1.5 s after statement presentation) and with variable duration determined per trial by reaction time to control for effects of reaction time on the neural response (Grinband *et al.*, 2008). Our main analysis included a model in which we conditionalized trials based on trial type (stereotype-consistent, stereotype-neutral or stereotype-violating trials in Study 1; stereotype-consistent, stereotype-violating, person-specific-confirming and person-specific-violating trials in Study 3). In our secondary analysis (Figure 2), we split each of the trial types included in the main analysis into two regressors, one in which participants provided a ‘Likely’ rating and one in which they provided an ‘Unlikely’ rating. We convolved events with a canonical hemodynamic response function and its temporal derivative and included additional covariates of no interest (session mean, no response trials, six motion parameters and their temporal derivative). The final first-level GLM was high-pass filtered at 128 s. Analyses were performed individually for each participant, and contrast images were subsequently entered into a second-level analysis treating participants as a random effect. We report activations that survived a threshold of *P* < 0.001 (uncorrected) at the voxel level and (cluster size) corrected to *P* < 0.05 at the cluster level using Monte Carlo simulations (1000 iterations) with the current imaging and analysis parameters (Slotnick, 2017).

#### Studies 2 and 4.

We analyzed choice data with logit generalized linear mixed models as implemented in the lme4 package version 1.1-14 (Bates *et al.*, 2015) for R version 3.4.2 (R Core Team, 2017). We used the probit link and included fixed effects for the intercept and the value difference between the two target types, as well as random effects for the intercepts for participants for the by-participant random slopes for the fixed effect of value difference. We calculated the PSE (and the related SEs) for the difference between the two target types for each study by the Delta Method as implemented in the MixedPsy package (Moscatelli *et al.*, 2012; Moscatelli and Balestrucci, 2017) for R. The Delta Method relies on responses to ‘all trials’ aggregated across participants in a generalized linear model to approximate the PSE with a Gaussian distribution (Moscatelli *et al.*, 2012) and to plot the cumulative distribution function. The model included the difference between the two target types on each trial as the predictor value and a binary outcome (stereotypical/knowledge-consistent option chosen) as the predicted value, with the probit link. To present individual-level data (Figure 3B and C), we calculated the PSE for each participant. This procedure resulted in some PSE values that exceeded the possible values in the current studies, as PSE models do not accurately reflect behavior when one choice option is rarely selected, as was the case for some participants. Therefore, we excluded participants for whom the calculated PSE value exceeded ±6 (*n* = 4–14 across studies).

## Supplementary Material

nsab081_Supp

## References

[R1] Adolphs R. (2009). The social brain: neural basis of social knowledge. *Annual Review of Psychology*, 60, 693–716. 10.1146/annurev.psych.60.110707.163514 PMC2588649 18771388

[R2] Allport G.W. (1954). *The Nature of Prejudice*. Oxford: Addison–Wesley.

[R3] Ames D.L., Fiske S.T. (2013). Outcome dependency alters the neural substrates of impression formation. *NeuroImage*, 83, 599–608. 23850465 10.1016/j.neuroimage.2013.07.001 PMC4478593

[R4] Amodio D.M., Devine P.G. (2006). Stereotyping and evaluation in implicit race bias: evidence for independent constructs and unique effects on behavior. *Journal of Personality and Social Psychology*, 91, 652–61. 17014291 10.1037/0022-3514.91.4.652

[R5] Asch S.E. (1946). Forming impressions of personality. *Journal of Abnormal and Social Psychology*, 41, 258–90. 10.1037/h0055756 20995551

[R6] Atzil S., Gao W., Fradkin I., et al. (2018). Growing a social brain. *Nature Human Behaviour*, 2, 624–36. 10.1038/s41562-018-0384-6 31346259

[R7] Ballard I., Miller E.M., Piantadosi S.T., et al. (2018). Beyond reward prediction errors: human striatum updates rule values during learning. *Cerebral Cortex*, 28, 3965–75. 29040494 10.1093/cercor/bhx259 PMC6685076

[R8] Bartra O., McGuire J.T., Kable J.W. (2013). The valuation system: a coordinate-based meta-analysis of BOLD fMRI experiments examining neural correlates of subjective value. *NeuroImage*, 76, 412–27. 23507394 10.1016/j.neuroimage.2013.02.063 PMC3756836

[R9] Bates D.M., Mächler M., Bolker B., et al. (2015). Fitting Linear Mixed-Effects Models Using lme4. *Journal of Statistical Software*, 67(1), 1–48.

[R10] Baumeister R.F., Leary M.R. (1995). The need to belong: desire for interpersonal attachments as a fundamental human motivation. *Psychological Bulletin*, 117, 497–529. 7777651

[R11] Berridge K.C. (2012). From prediction error to incentive salience: mesolimbic computation of reward motivation. *European Journal of Neuroscience*, 35, 1124–43. 10.1111/j.1460-9568.2012.07990.x PMC3325516 22487042

[R12] Bhanji J.P., Delgado M.R. (2014). The social brain and reward: social information processing in the human striatum. *Wiley Interdisciplinary Reviews: Cognitive Science*, 5, 61–73. 24436728 10.1002/wcs.1266 PMC3890330

[R13] Cacioppo J.T., Cacioppo S., Boomsma D.I. (2014). Evolutionary mechanisms for loneliness. *Cognition and Emotion*, 28, 1–22. 24067110 10.1080/02699931.2013.837379 PMC3855545

[R14] Chanes L., Wormwood J.B., Betz N., et al. (2018). Facial expression predictions as drivers of social perception. *Journal of Personality and Social Psychology*, 114, 380–96. 29369657 10.1037/pspa0000108 PMC5864287

[R15] Charpentier C.J., Bromberg-Martin E.S., Sharot T. (2018). Valuation of knowledge and ignorance in mesolimbic reward circuitry. *Proceedings of the National Academy of Sciences of the United States of America*, 115, E7255–64. 29954865 10.1073/pnas.1800547115 PMC6077743

[R16] Cloutier J., Gabrieli J.D.E., O’Young D., et al. (2011). An fMRI study of violations of social expectations: when people are not who we expect them to be. *NeuroImage*, 57, 583–8. 21569855 10.1016/j.neuroimage.2011.04.051

[R17] Cusack R., Papadakis N. (2002). New robust 3-D phase unwrapping algorithms: application to magnetic field mapping and undistorting echoplanar images. *NeuroImage*, 16, 754–64. 12169259 10.1006/nimg.2002.1092

[R18] Dale A.M. (1999). Optimal experimental design for event-related fMRI. *Human Brain Mapping*, 8, 109–14. 10524601 10.1002/(SICI)1097-0193(1999)8:2/3<109::AID-HBM7>3.0.CO;2-W PMC6873302

[R19] Darley J.M., Gross P.H. (1983). A hypothesis-confirming bias in labeling effects. *Journal of Personality and Social Psychology*, 44, 20–33.

[R20] Daw N.D., Gershman S.J., Seymour B., et al. (2011). Model-based influences on humans’ choices and striatal prediction errors. *Neuron*, 69, 1204–15. 21435563 10.1016/j.neuron.2011.02.027 PMC3077926

[R21] Deaner R.O., Khera A.V., Platt M.L. (2005). Monkeys pay per view: adaptive valuation of social images by rhesus macaques. *Current Biology*, 15, 543–8. 15797023 10.1016/j.cub.2005.01.044

[R22] Dunsmoor J.E., Kubota J.T., Li J., et al. (2016). Racial stereotypes impair flexibility of emotional learning. *Social Cognitive and Affective Neuroscience*, 11, 1363–73. 27107298 10.1093/scan/nsw053 PMC5015802

[R23] Eagly A.H., Karau S.J. (2002). Role congruity theory of prejudice toward female leaders. *Psychological Review*, 109, 573–98. 12088246 10.1037/0033-295x.109.3.573

[R24] Falben J., Tsamadi D., Golubickis M., et al. (2019). Predictably confirmatory: the influence of stereotypes during decisional processing. *Quarterly Journal of Experimental Psychology*, 72, 2437–51. 10.1177/1747021819844219 30931799

[R25] FeldmanHall O., Shenhav A. (2019). Resolving uncertainty in a social world. *Nature Human Behaviour*, 3, 426–35. 10.1038/s41562-019-0590-x PMC6594393 31011164

[R26] Festinger L. (1957). *A Theory of Cognitive Dissonance*, Vol. 2. Stanford, CA: Stanford university press.

[R27] Fiske S.T. Neuberg S.L. (1990). A continuum of impression formation, from category-based to individuating processes: influences of information and motivation on attention and interpretation. In: Zanna, M.P., editor. *Advances in Experimental Social Psychology*, New York, NY: Academic Press, 1–74.

[R28] Freeman J.B., Johnson K.L. (2016). More than meets the eye: split-second social perception. *Trends in Cognitive Sciences*, 20, 362–74. 27050834 10.1016/j.tics.2016.03.003 PMC5538856

[R29] Frimer J.A., Skitka L.J., Motyl M. (2017). Liberals and conservatives are similarly motivated to avoid exposure to one another’s opinions. *Journal of Experimental Social Psychology*, 72, 1–12.

[R30] Gawronski B. (2012). Back to the future of dissonance theory: cognitive consistency as a core motive. *Social Cognition*, 30, 652–68.

[R31] Gershman S.J. (2019). How to never be wrong. *Psychonomic Bulletin and Review*, 26, 13–28. 29799092 10.3758/s13423-018-1488-8

[R32] Glimcher P.W. (2011). Understanding dopamine and reinforcement learning: the dopamine reward prediction error hypothesis. *Proceedings of the National Academy of Sciences of the United States of America*, 108, 15647–54. 21389268 10.1073/pnas.1014269108 PMC3176615

[R33] Golman R., Hagmann D., Loewenstein G. (2017). Information avoidance. *Journal of Economic Literature*, 55, 96–135.

[R34] Greenwald A.G., Lai C.K. (2020). Implicit social cognition. *Annual Review of Psychology*, 71, 419–45. 10.1146/annurev-psych-010419-050837 31640465

[R35] Gregg A.P., Seibt B., Banaji M.R. (2006). Easier done than undone: asymmetry in the malleability of implicit preferences. *Journal of Personality and Social Psychology*, 90, 1–20. 16448307 10.1037/0022-3514.90.1.1

[R36] Grinband J., Wager T.D., Lindquist M., et al. (2008). Detection of time-varying signals in event-related fMRI designs. *NeuroImage*, 43, 509–20. 18775784 10.1016/j.neuroimage.2008.07.065 PMC2654219

[R37] Haber S.N., Knutson B. (2010). The reward circuit: linking primate anatomy and human imaging. *Neuropsychopharmacology*, 35, 4–26. 19812543 10.1038/npp.2009.129 PMC3055449

[R38] Hackel L.M., Doll B.B., Amodio D.M. (2015). Instrumental learning of traits versus rewards: dissociable neural correlates and effects on choice. *Nature Neuroscience*, 18, 1233–40. 26237363 10.1038/nn.4080

[R39] Hamilton D.L., Sherman S.J. (1996). Perceiving persons and groups. *Psychological Review*, 103, 336–55. 8637962 10.1037/0033-295x.103.2.336

[R40] Hare T.A., O’Doherty J.P., Camerer C.F., et al. (2008). Dissociating the role of the orbitofrontal cortex and the striatum in the computation of goal values and prediction errors. *Journal of Neuroscience*, 28, 5623–30. 18509023 10.1523/JNEUROSCI.1309-08.2008 PMC6670807

[R41] Harris L.T., Fiske S.T. (2010). Neural regions that underlie reinforcement learning are also active for social expectancy violations. *Social Neuroscience*, 5, 76–91. 20119878 10.1080/17470910903135825 PMC3891594

[R42] Hayden B.Y., Parikh P.C., Deaner R.O., et al. (2007). Economic principles motivating social attention in humans. *Proceedings of the Royal Society B*, 274, 1751–6. 17490943 10.1098/rspb.2007.0368 PMC2493582

[R43] Ho J., Tumkaya T., Aryal S., et al. (2019). Moving beyond P values: data analysis with estimation graphics. *Nature Methods*, 16, 565–6. 31217592 10.1038/s41592-019-0470-3

[R44] Huebner B. (2016). Implicit bias, reinforcement learning, and scaffolded moral cognition. In: Brownstein, M., Saul, J., editors. *Implicit Bias and Philosophy, Volume 1: Metaphysics and Epistemology*, New York, NY: Oxford University Press, 47–79.

[R45] Hughes B.L., Zaki J. (2015). The neuroscience of motivated cognition. *Trends in Cognitive Sciences*, 19, 62–4. 25640642 10.1016/j.tics.2014.12.006

[R46] Hutchinson J.B., Barrett L.F. (2019). The power of predictions: an emerging paradigm for psychological research. *Current Directions in Psychological Science*, 28, 280–91. 31749520 10.1177/0963721419831992 PMC6867616

[R47] Jepma M., Koban L., Doorn J., et al. (2018). Behavioural and neural evidence for self-reinforcing expectancy effects on pain. *Nature Human Behaviour*, 2, 838–55. 10.1038/s41562-018-0455-8 PMC6768437 31558818

[R48] Johnston L.C., Macrae C.N. (1994). Changing social stereotypes: the case of the information seeker. *European Journal of Social Psychology*, 24, 581–92.

[R49] Jost J., Hunyady O. (2003). The psychology of system justification and the palliative function of ideology. *European Review of Social Psychology*, 13, 111–53.

[R50] Knutson B., Westdorp A., Kaiser E., et al. (2000). FMRI visualization of brain activity during a monetary incentive delay task. *NeuroImage*, 12, 20–7. 10875899 10.1006/nimg.2000.0593

[R51] Kohli A., Blitzer D.N., Lefco R.W., et al. (2018). Using expectancy theory to quantitatively dissociate the neural representation of motivation from its influential factors in the human brain: an fMRI study. *NeuroImage*, 178, 552–61. 29751057 10.1016/j.neuroimage.2018.05.021

[R52] Krosch A.R., Amodio D.M. (2019). Scarcity disrupts the neural encoding of Black faces: a aocioperceptual pathway to discrimination. *Journal of Personality and Social Psychology*, 117, 859–75. 31233317 10.1037/pspa0000168

[R53] Lefebvre G., Lebreton M., Meyniel F., et al. (2017). Behavioural and neural characterization of optimistic reinforcement learning. *Nature Human Behaviour*, 1, 1–9.

[R54] Lin A., Adolphs R., Rangel A. (2012). Social and monetary reward learning engage overlapping neural substrates. *Social Cognitive and Affective Neuroscience*, 7, 274–81. 21427193 10.1093/scan/nsr006 PMC3304477

[R55] Lockwood P.L., Apps M.A.J., Chang S.W.C. (2020). Is there a ‘social’ brain? Implementations and algorithms. *Trends in Cognitive Sciences*, 24, 802–13. 32736965 10.1016/j.tics.2020.06.011 PMC7501252

[R56] Lord C.G., Ross L., Lepper M.R. (1979). Biased assimilation and attitude polarization: the effects of prior theories on subsequently considered evidence. *Journal of Personality and Social Psychology*, 37, 2098–109.

[R57] Macrae C.N., Bodenhausen G.V. (2000). Social cognition: thinking categorically about others. *Annual Review of Psychology*, 51, 93–120. 10.1146/annurev.psych.51.1.93 10751966

[R58] Matthews G.A., Tye K.M. (2019). Neural mechanisms of social homeostasis. *Annals of the New York Academy of Sciences*, 1457, 5–25. 30875095 10.1111/nyas.14016 PMC7593988

[R59] Mende-Siedlecki P., Cai Y., Todorov A. (2013). The neural dynamics of updating person impressions. *Social Cognitive and Affective Neuroscience*, 8, 623–31. 22490923 10.1093/scan/nss040 PMC3739907

[R60] Morgenroth T., Ryan M.K. (2020). The effects of gender trouble: an integrative theoretical framework of the perpetuation and disruption of the gender/sex binary. *Perspectives on Psychological Science*. 2020. 10.1177/1745691620902442 10.1177/1745691620902442 PMC8564221 32375012

[R61] Moscatelli A., Mezzetti M., Lacquaniti F. (2012). Modeling psychophysical data at the population-level: the generalized linear mixed model. *Journal of Vision*, 12, 26. 10.1167/12.11.26 23104819

[R62] Moscatelli A., Balestrucci P. (2017). Psychophysics with R: the R package MixedPsy.

[R63] Moss-Racusin C.A., Johnson E.R. (2016). Backlash against male elementary educators. *Journal of Applied Social Psychology*, 46, 379–93.

[R64] Niv Y., Schoenbaum G. (2008). Dialogues on prediction errors. *Trends in Cognitive Sciences*, 12, 265–72. 18567531 10.1016/j.tics.2008.03.006

[R65] O’Doherty J.P. (2014). The problem with value. *Neuroscience and Biobehavioral Reviews*, 43, 259–68. 24726573 10.1016/j.neubiorev.2014.03.027 PMC4332826

[R66] Olszanowski M., Kaminska O.K., Winkielman P. (2018). Mixed matters: fluency impacts trust ratings when faces range on valence but not on motivational implications. *Cognition and Emotion*, 32, 1032–51. 29057707 10.1080/02699931.2017.1386622

[R67] Otten M., Seth A.K., Pinto Y. (2017). A social Bayesian brain: how social knowledge can shape visual perception. *Brain and Cognition*, 112, 69–77. 27221986 10.1016/j.bandc.2016.05.002

[R68] Oyserman D. Yan V.X. (2019). Making meaning: a culture-as-situated-cognition approach to the consequences of cultural fluency and disfluency. In: Cohen, D., Kitayama, S., editors. *Handbook of Cultural Psychology*, New York, NY: Guilford Press, 536–65.

[R69] Patil I. (2021). Visualizations with statistical details: The ‘ggstatsplot’ approach. *Journal of Open Source Software*, 6(61), 3167.

[R70] Peters J., Büchel C. (2010). Neural representations of subjective reward value. *Behavioral Bran Research*, 213, 135–41. 10.1016/j.bbr.2010.04.031 20420859

[R71] Peters U. (2020). What is the function of confirmation bias? *Erkenntnis*. 10.1007/s10670-020-00252-1

[R72] Phelan J.E., Rudman L.A. (2010). Reactions to ethnic deviance: the role of backlash in racial stereotype maintenance. *Journal of Personality and Social Psychology*, 99, 265–81. 20658843 10.1037/a0018304

[R0073a] R Core Team (2017). R: A language and environment for statistical computing. R Foundation for Statistical Computing, Vienna, Austria. https://www.R-project.org/.

[R73] Roese N.J. Sherman J.W. (2007). Expectancy. In: Higgins, E.T., Kruglanski, A.W., editors. *Social Psychology: Handbook of Basic Principles*, New York, NY: The Guilford Press, 91–115.

[R74] Rudman L.A., Moss-Racusin C.A., Phelan J.E., et al. (2012). Status incongruity and backlash effects: defending the gender hierarchy motivates prejudice against female leaders. *Journal of Experimental Social Psychology*, 48, 165–79.

[R75] Ruissen M.I., Overgaauw S., De Bruijn E.R.A. (2018). Being right, but losing money: the role of striatum in joint decision making. *Scientific Reports*, 8, 1–11. 29712917 10.1038/s41598-018-24617-3 PMC5928107

[R76] Satterthwaite T.D., Ruparel K., Loughead J., et al. (2012). Being right is its own reward: load and performance related ventral striatum activation to correct responses during a working memory task in youth. *NeuroImage*, 61, 723–9. 22484308 10.1016/j.neuroimage.2012.03.060 PMC3358426

[R77] Schultz W. (2000). Multiple reward signals in the brain. *Nature Reviews: Neuroscience*, 1, 199–207. 11257908 10.1038/35044563

[R78] Schwarz K.A., Sprenger C., Hidalgo P., et al. (2019). How stereotypes affect pain. *Scientific Reports*, 9, 8626. 10.1038/s41598-019-45044-y PMC6565709 31197222

[R79] Sescousse G., Caldú X., Segura B., et al. (2013). Processing of primary and secondary rewards: a quantitative meta-analysis and review of human functional neuroimaging studies. *Neuroscience and Biobehavioral Reviews*, 37, 681–96. 23415703 10.1016/j.neubiorev.2013.02.002

[R80] Sharot T., Guitart-masip M., Korn C.W., et al. (2012). How Dopamine enhances an optimism bias in humans. *Current Biology*, 22, 1477–81. 22795698 10.1016/j.cub.2012.05.053 PMC3424419

[R81] Singmann H., Bolker B., Westfall J., et al. (2018). Afex: analysis of factorial experiments. https://CRAN.R-project.org/package=afex.

[R82] Slotnick S.D. (2017). Cluster success: fMRI inferences for spatial extent have acceptable false-positive rates. *Cognitive Neuroscience*, 8, 150–5. 28403749 10.1080/17588928.2017.1319350

[R83] Smith E.R., Miller D.A., Maitner A.T., et al. (2006). Familiarity can increase stereotyping. *Journal of Experimental Social Psychology*, 42, 471–8.

[R84] Stern C., West T.V., Rule N.O. (2015). Conservatives negatively evaluate counterstereotypical people to maintain a sense of certainty. *Proceedings of the National Academy of Sciences of the United States of America*, 112, 15337–42. 26621712 10.1073/pnas.1517662112 PMC4687596

[R85] Stern C., Rule N.O. (2018). Physical androgyny and categorization difficulty shape political conservatives’ attitudes toward transgender people. *Social Psychological and Personality Science*, 9, 24–31.

[R86] Sullivan J. (2019). The primacy effect in impression formation: some replications and extensions. *Social Psychological and Personality Science*, 10, 432–9.

[R87] Tamir D.I., Mitchell J.P. (2012). Disclosing information about the self is intrinsically rewarding. *Proceedings of the National Academy of Sciences of the United States of America*, 109, 8038–43. 22566617 10.1073/pnas.1202129109 PMC3361411

[R88] Tamir D.I., Thornton M.A. (2018). Modeling the predictive social mind. *Trends in Cognitive Sciences*, 22, 201–12. 29361382 10.1016/j.tics.2017.12.005 PMC5828990

[R89] Tay L., Tan K., Diener E., et al. (2013). Social relations, health behaviors, and health outcomes: a survey and synthesis. *Applied Psychology: Health and Well-Being*, 5, 28–78. 23281315 10.1111/aphw.12000

[R90] Theriault J.E., Young L., Barrett L.F. (2021). The sense of should: a biologically-based framework for modeling social pressure. *Physics of Life Reviews*, 36, 100–36. 32008953 10.1016/j.plrev.2020.01.004 PMC8645214

[R91] Tibboel H., De Houwer J., Bockstaele B.V. (2015). Implicit measures of ‘wanting’ and ‘liking’ in humans. *Neuroscience and Biobehavioral Reviews*, 57, 350–64. 26432503 10.1016/j.neubiorev.2015.09.015

[R92] Tricomi E., Fiez J.A. (2008). Feedback signals in the caudate reflect goal achievement on a declarative memory task. *NeuroImage*, 41, 1154–67. 18445531 10.1016/j.neuroimage.2008.02.066 PMC2487673

[R93] Ullsperger M., von Cramon D.Y. (2003). Error monitoring using external feedback: specific roles of the habenular complex, the reward system, and the cingulate motor area revealed by functional magnetic resonance imaging. *Journal of Neuroscience*, 23, 4308–14. 12764119 10.1523/JNEUROSCI.23-10-04308.2003 PMC6741115

[R94] Uusberg A. Suri G. Dweck C.S. , et al. (2019). Motivation: a valuation systems perspective. In: Neta, M., Haas, I.J., editors. *Emotion in the Mind and Body. Nebraska Symposium on Motivation*, Vol. 66, Cham: Springer, 161–92.

[R95] Welborn B.L., Hong Y., Ratner K.G. (2020). Exposure to negative stereotypes influences representations of monetary incentives in the nucleus accumbens. *Social Cognitive and Affective Neuroscience*, 15, 347–58. 32248234 10.1093/scan/nsaa041 PMC7235954

[R96] Wu H., Luo Y., Feng C. (2016). Neural signatures of social conformity: a coordinate-based activation likelihood estimation meta-analysis of functional brain imaging studies. *Neuroscience and Biobehavioral Reviews*, 71, 101–11. 27592151 10.1016/j.neubiorev.2016.08.038

[R97] Wyer N.A. (2010). You never get a second chance to make a first (implicit) impression: the role of elaboration in the formation and revision of implicit impressions. *Social Cognition*, 28, 1–19.

[R98] Yon D., de Lange F.P., Press C. (2019). The predictive brain as a stubborn scientist. *Trends in Cognitive Sciences*, 23, 6–8. 30429054 10.1016/j.tics.2018.10.003

