# Supplementary material for: Confirmation of interpersonal expectations is intrinsically rewarding
Source: Soc Cogn Affect Neurosci. 2021 Jul 7;16(12):1276–87. doi: 10.1093/scan/nsab081 (PMC8717061; doi:10.1093/scan/nsab081)
Supplement: nsab081_Supp [file nsab081_supp.zip › scan-21-005-File005.docx]

Supplementary Materials for

**Confirmation of interpersonal expectations is intrinsically rewarding**

Niv Reggev, Anoushka Chowdhary and Jason P. Mitchell, Harvard University.

Corresponding author: Niv Reggev

Email: [reggevn@bgu.ac.il](mailto:reggevn@bgu.ac.il)

**Supplementary Materials include:**

Supplementary text

SM References

Figures S1 to S4

Tables S1 to S6

**Supplementary Materials: Materials and Methods**

Below we provide a detailed description of all the materials and methods used, including all measures and data exclusions.

*Participants*

**Studies 1 and 3.** Twenty-eight individuals participated in Study 1 (mean age: 21.85, S.D.: 2.95, range: 18–30, 21 females, 15 Caucasian, 4 Hispanic, 4 mixed, 3 Asian, 2 African American) and 30 individuals participated in Study 3 (mean age: 22.37, S.D.: 2.62, range: 18–30, 17 females, 14 Caucasian, 7 Asian, 4 African American, 3 mixed, 1 Hispanic, 1 did not self-identify). Participants were recruited from Harvard University and its surroundings using Harvard’s Psychology Study Pool website. Additional participants were excluded due to technical issues (1 participant from Study 3), lack of response to more than 20% of trials (2 participants from each study), or excessive movement (more than 1 mm – half-width of the acquired voxel size) in more than one functional run in the scanner (1 participant in Study 1, 3 participants in Study 3). All participants were healthy, right-handed, native English speakers with normal or corrected-to-normal vision and no history of neurological or psychiatric conditions. Participants were compensated with $65 in Study 1 and $55 in Study 3. We determined supplemental compensation (up to $30) based on participants’ performance in the monetary incentive delay task (see below). Pilot versions of the imaging tasks were conducted outside the scanner to ensure the functionality of the task.

**Studies 2 and 4.** Three-hundred-ninety-six individuals from Amazon Mechanical Turk completed Study 2 (in Study 2a, 198 out of 216 individuals who started the study; in Study 2b, 198 out of 223 individuals). Study 2a was conducted in March 2018, and Study 2b was conducted in April 2018. Four hundred seventy individuals from Prolific Academic completed Studies 4a and 4b in January 2019 (235 in each study; a total of 503 participants started the study). All participants were 18 years old or older, had an approval rate of 95% or higher, held a US nationality, and participated only in one of the studies. In addition, participants from Amazon Mechanical Turk were required to have completed at least 100 tasks to be eligible for Study 2. The final sample size we report in the main text incorporates the following pre-registered exclusion criteria for participants who (1) answered incorrectly at least two of the manipulation or attention checks, (2) completed the survey in more than 20 minutes or less than 2.5 standard deviations below the sample mean duration, (3) failed to answer more than 15% of the survey, (4) provided similar ratings (on the 0–100 scale) on all trials; specifically, participants whose standard variation of the rating was two standard deviations below the sample mean standard deviation (not applied in Study 2a), (5) provided an identical response in all 2AFC trials (applied only in Study 4) and (6) indicated in their debriefing that they had understood the goal of the task and had acted per this understanding at any point during the task. Participants in all studies were similarly distributed on gender (Study 2a: 51.72% males, 45.98% females; Study 2b: 50.3% males, 48.52% females; Study 4a: 51.32% males, 46.56% females; Study 4b: 41.86% males, 56.98% females; across studies 1%–3% of participants self-identified as gender-nonconforming or other identification), age (Study 2a: Mean [S.D.]: 37.47 [11.75]; Study 2b: 35.13 [10.04]; Study 4a: 31.22 [10.32]; Study 4b: 31.99 [10.97]) and ethnicity (Study 2a: 78.16% White/European American; Study 2b: 74.56% White/European American; Study 4a: 69.84% White/European American; Study 4b: 69.19% White/European American). Study 4 measured political orientation on a 1–9 scale (see below). We observed no differences in this measure between Study 4a and Study 4b (Study 4a: 3.79 [2.13], Study 4b: 3.45 [1.96]).

*Stimuli*

All studies included face-statement pairs. We selected faces from the 10k US Adult Faces Database (a large-scale database of natural face photographs of the U.S. adult population (Bainbridge *et al.*, 2013)). The current investigation focused on Caucasian faces to avoid intrusion of racial or intersectional stereotypes. We further restricted the faces to be moderately memorable (0.4 – 0.6 hit rate in the 10k database, computed over an average of 81.7 people per photo) and excluded faces with any distinguishable accessories (e.g., hats, big necklaces, etc.). This resulted in a total of 204 faces, 102 per gender. Three additional photos per gender were used in the practice sessions. All face pictures had neutral to mildly positive expressions. We resized all pictures to 240 by 256 pixels and presented them in color on a gray background (see Figure S1). For Studies 3 and 4, we also included images portraying the faces of Barack Obama and Donald Trump (a single image per leader). These images were taken from open access sources and cropped to be identical in dimensions to the rest of the face stimuli.

We generated gender-stereotype-relevant statements in the form of individual preference, trait, behavior, or profession (e.g., “Loves taking risks,” “Doesn’t cry,” “Swears a lot,” “Is a truck driver,” etc.; see https://osf.io/tgja3). We piloted the verbal statements in a two-phase procedure using non-overlapping groups of participants that did not participate in the reported studies. In the first session, 23 participants rated 160 gender-related statements generated by the authors based on various sources (e.g., Prentice and Carranza, 2002). Participants saw each statement once and indicated how typical the characteristic was for a specific gender on a visual scale of 0 (= “very untypical”) to 100 (= “very typical”; the scale had no other tick marks). Each participant rated each statement either for men or for women, never for both, randomly determined per participant. Participants were instructed to base their ratings on how they thought the average person would respond. Based on the ratings from this first phase, we selected 136 gender-related statements (68 per gender) for which the average rating was at least 65 for one of the genders and less than 35 for the other gender. In a second session of the pilot testing, we verified the stereotypicality of these statements with a total of 78 participants who used the same rating procedure to rate the selected 136 statements and an additional 68 gender-neutral statements (e.g., “Exercises regularly,” “Thinks positively,” “Is a TV reporter”). The statements included in the studies contained 2–9 words (mean: 4.69, S.D.: 1.44; no difference between experimental conditions, *p* > 0.2) and 9–45 characters (mean: 26.68, S.D.: 7.69; *p* > 0.18).

We utilized a similar procedure for leader-relevant statements. First, we generated 160 statements based on common knowledge about the selected leaders. We constructed the statements such that a statement consistent with knowledge about one leader would violate knowledge about the other leader. We piloted these statements with 243 Amazon Mechanical Turk participants. Each participant saw a randomly selected subset of 40 or 60 statements and rated them on two scales: “how likely is the statement to be attributed to Barack Obama” and “how likely is the statement to be attributed to Donald Trump” on a visual scale of 0 (= “very unlikely”) to 100 (= “very likely”; the scale had no other tick marks). Participants were instructed to base their ratings on how they thought the average person would respond. Each statement had an average of 81.75 raters (S.D.: 6.23; range: 67–100). We selected the 120 statements that differed the most between ratings for the two leaders. Overall, the statements contained 2–9 words (mean: 4.65, S.D.: 1.52; no difference between experimental conditions, *p* > 0.5) and 11–45 characters (mean: 28.33, S.D.: 8.43; *p* > 0.5).

*Behavioral procedure*

**Studies 1 and 3.** The impression formation task included 204 face-statement pairs in Study 1 and 240 face-statement pairs in Study 3. To create stereotype-consistent and stereotype-violating pairs, we yoked half of the 136 stereotypical statements (120 statements in Study 3) with faces from the gender corresponding to the stereotype and the other half with faces from the mismatching gender (yoking of statements was randomized across participants; e.g., the statement “Doesn’t cry” could be paired with a male face for one participant and with a female face for another participant to create a stereotype-consistent or a stereotype-violating pair, respectively). Gender-neutral statements (presented only in Study 1; 68 statements) were randomly and evenly yoked with male and female faces. We randomized the specific face identity paired with each statement across participants. We used a similar procedure to create leader-specific pairs (60 statements per leader), with the exception that a single photo was used per leader.

*Impression Formation Task.* Each trial in the Impression Formation task (see Figure S1) started with a statement (describing a neutral, stereotypically male, or stereotypically female characteristic in Study 1; stereotypically male, stereotypically female, Obama-specific, or Trump-specific in Study 3) presented for 1.5 seconds. Then, a face joined the statement to form a face-statement pair that was either consistent or inconsistent (or, in Study 1, neutral) with gender stereotypes or (in Study 3) person-specific knowledge. The pair appeared on screen for 4 seconds for a total of 5.5 seconds per trial in Study 1 and 3.5 seconds for a total of 5 seconds in Study 3. Each trial ended with a 0.5-second fixation crosshair. For each pair, participants used their left hand to indicate how likely the presented target was to be described by the specific characteristic using a 4-point scale (1- “very unlikely”’; 4 - “very likely”). Participants were to provide a response while the pair appeared on screen.

Each of four functional runs included 51 (in Study 1) or 60 (in Study 3) unique face-statement pairs (17 per condition in Study 1, 15 per condition in Study 3). In Study 3, we presented statements in mini-blocks by knowledge domain (stereotypes versus person-specific), two blocks per type per run; each mini-block contained 15 trials. The order of the mini-blocks was randomly determined within each run with a limitation that consecutive blocks never displayed the same type of content. To optimize the estimation of the event-related fMRI response, we intermixed conditions in a pseudorandom order and separated trials by a variable interstimulus interval (Study1: 0–9 seconds, mean: 3.03, S.D.: 2.25; Study 3: 0–7 seconds, mean: 1.24, S.D.: 1.84). We used OptSeq2 (Dale, 1999) to generate sequences optimized for the efficiency of a 3-conditions design for a first-order counterbalanced event sequence in Study 1 and the efficiency of the (expectancy-consistent versus expectancy-violating) contrast for a first-order counterbalanced event sequence in Study 3. Of these sequences, we selected six sequences that contained no more than 5 consecutive events of the same condition (separate sequences were generated and selected per Study). We randomly assigned (with replacement) an event sequence for each functional run to avoid spurious results attributable to differences between conditions in one specific event sequence (Mumford *et al.*, 2014). Within conditions, trials were presented in random order. To facilitate familiarization with the task, participants completed a brief practice session before entering the MRI machine. This practice session included 6 statement-face pairs in Study 1 and 12 pairs in Study 3; the stimuli used in the practice session were not used in any other phase of the experiment.

*Monetary Incentive Delay (MID) task.* After completing the impression formation task, participants in Studies 1 and 3 completed the Monetary-Incentive Delay (MID) task (Knutson *et al.*, 2000) to allow us to localize brain regions associated with the processing of rewarding stimuli in a non-social context. Participants were not informed about this task before its execution to prevent them from forming an association between the main task and reward processing.

The MID task included a series of trials in which participants attempted to respond, via a button press, to a briefly presented target (a white rectangle) (see Figure S1B). Each trial started with a cue (a blue circle or a green circle) shown for 0.5 seconds. The green circle predicted a modest monetary reward ($1) upon a successful response to the target, whereas the blue circle predicted no reward. Nevertheless, participants were instructed to respond to both cue types. Cues were followed by a delay interval randomly varying in duration between 2 and 2.5 seconds. The target was then briefly presented for a duration varied between 130 and 350 milliseconds. Duration varied as a function of the participants’ performance. Specifically, we implemented a 2-down 1-up staircase procedure to create a level of difficulty that would allow participants to successfully respond to the target on two-thirds of the trials. This algorithm succeeded; on average, participants were rewarded on approximately 20 of the 30 trials (Study 1 mean: 20.26; Study 3: 20.21). At the end of each trial, participants saw the amount of money they had earned on that trial along with the total amount they had earned during the task up to that point (presented for 0.5 seconds). The task included 45 trials (with 30 green cue trials). We added participants' gains in this task to their overall compensation.

*Memory Test.* Once outside the scanner, participants completed a surprise associative memory task that will be reported elsewhere (Reggev & Mitchell, in preparation). Briefly, none of the results reported in the current manuscript were affected by including memory in the analyses.

*Additional measures.* Next, participants completed several individual differences and explicit attitudes scales measuring beliefs about sexism (Ambivalent Sexism Inventory - ASI) (Glick and Fiske, 1996), social dominance orientation (SDO) (Ho *et al.*, 2015), motivation to control sexism (MCS) (Klonis *et al.*, 2005) and need for cognitive closure (NFC) (Kruglanski *et al.*, 1993). The order of the scales was randomized between participants. We included these scales to facilitate future individual differences analyses. Individual differences scores were not used in the current manuscript in any of the analyses due to insufficient power and are reported solely for full disclosure’s sake.

Finally, participants in Study 1 indicated the extent to which they thought the different statements presented during the impression formation task were associated with women and men using the procedure used for piloting the statements. We presented each statement with the gender to which it was yoked in the impression formation task. Participants had up to 10 seconds per trial and were told that they should base their judgments on their own beliefs about men and women rather than what the "average" person in the population thinks.

After completing these tasks, participants provided demographic details (age, self-identified gender, and self-identified race in an open response format, and in Study 3, political affiliation on a 1–9 scale, 1 = “extremely liberal” and 9 = “extremely conservative”). Then, we probed participants for their understanding of the goal of the study and asked whether they had suspected a memory test. Lastly, we paid and fully debriefed them.

*Imaging procedure*

Images were collected with a 3T Siemens Prisma scanner system (Siemens Medical Systems, Erlangen, Germany) using a 64-channel radiofrequency head coil. Stimuli were projected onto a screen at the end of the magnet bore that participants viewed via a mirror mounted on the head coil. Stimulus presentation was controlled by PsychoPy v1.84.2 (Peirce, 2007) running under Windows 7. Prior to entering the scanner, participants were extensively briefed by one of the authors about potential movements that can occur in the scanner and ways to mitigate them. Participants were then set up in the scanner, head first and supine in the scanner bore, with a response box in their left hand. Foam cushions were placed within the head coil to minimize head movements. First, high-resolution anatomical images were acquired using a T1-weighted 3D MPRAGE sequence (TR = 2200 msec, TI = 1100 msec, acquisition matrix = 256 × 256 × 176, flip angle = 7, voxel size = 1 × 1 × 1 mm^3^). Second, a fieldmap was acquired in the same plane as the functional images (see below) to correct for inhomogeneities in the magnetic field (Cusack and Papadakis, 2002). Next, whole-brain functional images were collected using a simultaneous multi-slice (multiband) T2*-weighted gradient echo sequence, sensitive to BOLD contrast, developed at the Center for Magnetic Resonance Research (CMRR) at University of Minnesota (Feinberg *et al.*, 2010; Moeller *et al.*, 2010; Xu *et al.*, 2013) (TR = 2000 msec, TE = 30 msec, voxel size = 2 × 2 × 2 mm^3^, 75 slices auto-aligned to -25 degrees of the AC-PC line, image matrix = 104 × 104, FOV = 208 * 208 mm^2^, flip angle = 75º, GRAPPA acceleration factor = 2, multiband factor = 3, phase encoding direction = A -> P). After a brief practice run (identical in content to the practice session completed before entering the scanner), participants completed four impression formation task runs consisting of 229 volumes each in Study 1 and 245 volumes each in Study 3; all runs were complemented by two additional dummy scans and an initial period of approximately 26 seconds dedicated to references for the GRAPPA procedure. The first four volumes from each run (i.e., in addition to dummy scans) were discarded to ensure T1 equilibrium. The last 5 volumes from each run always included a crosshair fixation to ensure the appropriate estimation of the hemodynamic function for the last events in the run. Finally, participants completed the MID task in a single run consisting of 110 volumes using identical parameters to those mentioned above.

*Imaging analysis*

We processed and analyzed the fMRI data using SPM12 version 6225 (Wellcome Department of Cognitive Neurology, London, UK) on a 2015b MATLAB platform (Mathworks, Natick, MA, USA). Functional data were corrected for differences in acquisition time between slices, corrected for inhomogeneities in the magnetic field using the fieldmap (Cusack and Papadakis, 2002), realigned to the first image to correct for head movement using a 2^nd^ degree B-spline interpolation, unwarped to account for residual movement-related variance using a 4^th^ degree B-spline interpolation and co-registered with each participant’s anatomical data. Then, the functional data were transformed into standard anatomical space (2 mm isotropic voxels) based on the ICBM152 brain template (Montreal Neurological Institute). Normalized data were spatially smoothed (6 mm full-width at half-maximum, FWHM) using a Gaussian Kernel. In addition to the GLM models reported in the main text, in Study 1, we also examined a model in which the stereotypicality of trials was modeled continuously rather than with the dichotomous binning approach. Specifically, this additional model included two regressors - one for trials including a woman’s face and another for trials including a man’s face. We included a separate parametric modulator for each regressor to model the extent of the stereotypicality of the statement included in that trial based on our pilot ratings. For example, the statement “Can lift heavy things” was rated as related more to men than to women in our pilot studies with a 28-points difference on the 0–100 scale. Consequently, the parametric modulation value was 0.28 for trials in which this statement was presented with a man’s face, and -0.28 for trials in which this statement was presented with a woman’s face. Similar to the models reported in the main text, in this additional model we convolved events with a canonical hemodynamic response function and its temporal derivative and included additional covariates of no interest (session mean, no response trials, six motion parameters, and their temporal derivative).

*Regions of interest (ROIs)*

We used two complementary approaches to localize regions involved in the processing of rewards. For independently defined ROIs, we defined 8 mm spheres around peak coordinates drawn from a recent meta-analysis (Bartra *et al.*, 2013). Specifically, we utilized the peaks of the region identified as supporting the processing of both monetary and primary incentives: bilateral ventral striatum (x=-6, y=10, z=-6 and x=10, y=12, z=-6).

To functionally locate these regions, we examined the MID task to identify voxels that responded more to rewarded trials (i.e., trials in which participants successfully responded to the target) than to no-reward trials (i.e., trials in which no reward was available). Whole-brain corrected clusters (using the procedure described in the main text) were defined as independent ROIs.

As we had no prediction about the laterality of the hypothesized effects, we collapsed across hemispheres to create a single ROI. We extracted and averaged parameter estimates across voxels and analyzed them with planned contrasts in a repeated-measures ANOVA context using p<0.05 as a threshold.

**Studies 2 and 4.** In each trial, participants chose one of two decks of cards (see Figure S2). We instructed participants to choose based on their preferences regarding the information they had available for each trial. Participants had two sources of information to rely on. First, each deck was associated with a small monetary payoff (ranging from $0.03 to $0.09 in 2 cents increments). Participants were told that a subset of their choices (5 trials in Study 2, 7 trials in Study 4) would be added to their final compensation for the study. Payoff amounts for each choice varied across trials (and were occasionally equal). Payoff disparities (i.e., the differences in monetary values associated with each deck) followed a quasi-gaussian distribution, such that the most extreme disparities (6 cents in favor of one deck or the other) always appeared the least, twice (Study 2) or thrice (Study 4) for each participant. As disparities grew smaller, they gradually became more frequent, with the zero disparity trials (i.e., equivalent monetary values for the two sets) appearing most frequently (5 times in Study 2, 6 times in Study 4). Second, each deck was associated with a specific label (“Typical” versus “Atypical” in Study 2, “Common” versus “Uncommon” in Study 4) that determined the content presented when that deck is selected (stereotypical or counter-stereotypical targets in Study 2, knowledge-consistent or knowledge-violating targets in Study 4). After making their selection, participants saw a face-statement pair corresponding to their choice. For example, if a participant in Study 2 selected the “Typical” deck, they would be presented with a stereotypical target (e.g., in Study 2a, a male associated with the statement “CEO of a big company”). Participants then indicated how likely that target was to possess that characteristic on a visual scale of 0 (= “Not at all likely”) to 100 (= “Very likely”; the scale had no other tick marks). The location of the specific labels (right deck versus left deck) was counterbalanced between participants. Study 2a included only faces of men, Study 2b included only faces of women, Study 4a included only the face of Barack Obama, and Study 4b included only the face of Donald Trump.

All studies included a demo trial, 4 practice trials, and 25 deck selection trials (32 trials in Study 4). We also included several catch trials to detect if participants were responding without considering the statement presented. The catch trials prompted the participants to slide the bar to the right tick mark or the left tick mark. Participants had up to 4 seconds to select a card and up to 5 seconds to rate an individual. Participants that did not respond fast enough to more than 20% of the trials got their survey terminated midway through the task. The specific amounts associated with each trial and the face-statement pairs presented per trial were randomized. Following these trials, participants were presented with 4 final manipulation-check trials in which they were asked to select the card with the higher value.

After completing the main task, participants responded to the individual difference scales mentioned above – ASI, SDO, MCP, and NFC. The order of the scales was randomized between participants. No significant correlations between the Point of Subjective Equivalence (PSE) and the individual scores on these scales were consistently detected across studies. Finally, participants supplied demographic information (including age, self-reported gender identity, self-reported race with multiple answers enabled, and whether they were born in the US). Study 4 also probed participants’ political affiliation (as in Study 3), how much they liked Barack Obama and how much they liked Donald Trump on two separate 0–100 scales. Then, we probed for participants’ intuitions about the goal of the task and fully debriefed them.

**Supplementary Materials Results**

*Behavioral analyses: Neuroimaging studies*

Table S1 summarizes participants’ ratings and reaction times in the impression formation task in Study 1. We analyzed rating data with mixed models as implemented in the lme4 package version 1.1-14 (Bates *et al.*, 2014) and the ‘ordinal’ package version 2018.4-19 (Christensen, 2018) for R version 3.4.2 (R Core Team, 2017). As the behavioral data obtained in the impression formation task were ordinal, we analyzed them using cumulative link mixed models (CLMM) with the logit link. To avoid the transformation of raw reaction time data, we used generalized linear models (gLMMs) with the inverse Gaussian identity link (Lo and Andrews, 2015). We included random effects for the intercepts for participants and statements, as well as by-participant random slopes for the fixed effect of stereotypicality. Trials that elicited no response (<1.5% of all trials; no difference between conditions) were excluded from all analyses.

To examine whether our stereotype-consistent and stereotype-violating targets were indeed perceived differently by our participants in Study 1, we tested the effect of our a-priori categorization on behavioral ratings in a CLMM. Stereotypicality was dummy coded with stereotype-neutral trials as the intercept, and behavioral ratings were centered on 0. Overall stereotypicality affected ratings, as indicated by leave-one-out model comparison, comparing our model to an intercept-only model (*χ^2^*_(2)_ = 44.45, *p* < 0.001). The manipulation worked as anticipated: Stereotype-consistent targets received higher ratings than stereotype-neutral targets (*β*±*SE* = 0.42±0.14, *Z* = 2.99, *p* = 0.003), whereas stereotype-violating targets received lower ratings (*β*±*SE* = -1.22±0.15, *Z* = -8.31, *p* < 0.001).

Stereotypicality did not have a main effect on reaction time for the behavioral ratings (*χ^2^*_(2)_ = 0.35, *p* > 0.8). However, reaction time did vary by the interaction of stereotypicality and behavioral ratings, as indicated by comparing a model with an interaction term to a model without it, *χ^2^*_(2)_ = 133.88, *p* < 0.001); participants were slower to provide responses that were not in line with our a-priori definitions (e.g., indicating that a woman is very likely to be a firefighter or saying that a man is very unlikely to be a CEO); see Table S1 for full descriptive results.

Behavioral results in Study 3 generally replicated the behavioral results we obtained in Study 1 (see Table S3). The outcome of the expectation (whether the target was consistent with or violated the expectation) significantly affected participants’ ratings (*χ^2^*_(1)_ = 73.61, *p* < 0.001). Domain (stereotype-based or person-specific-based knowledge) also affected participants’ ratings (*χ^2^*_(1)_ = 27.58, *p* < 0.001). Interestingly, participants distributed their expectation-based responses differently between the domains (outcome by domain interaction: *χ^2^*_(1)_ = 862.52, *p* < 0.001). Participants used more extreme outcome-congruent ratings for person-specific expectations compared to stereotype-based expectations (see Table S3).

Similar to Study 1, participants’ reaction time did not differ between expectation-consistent or expectation-violating trials (*t =* -0.29, *p =* 0.77), nor between person-specific or stereotype-based expectations (*t* = 1.89, *p* = 0.059). Comparable to Study 1, we observed a significant interaction between outcome and behavioral ratings (*t* = -15.11, *p* < 0.001), an interaction that was qualified by a 3-way interaction with domain (*t* = 6.36, *p* < 0.001). To interpret this interaction, we analyzed responses separately for the two knowledge domains. In both knowledge domains, participants responded faster when their responses were in line with our a-priori definitions (*t* = -9.72 and *t* = -13.81 for stereotype- and person-specific-based expectations, respectively; *p’s* < 0.001).

*Neuroimaging*

In Study 3, in addition to the main findings (see Figure 1 and Table S4), the design allowed us to compare the effects of expectation-consistency with different specific targets, namely, men, women, Trump or Obama. Analyzing activity in the NAcc in a 2 (expectation result) X 2 (content domain) X 2 (specific target) ANOVA yielded, in addition to the main effect of expectation consistency (*F*_(1,29)_ = 21.41, *p* < 0.0001, *η^2^_p_* = 0.42 [0.19–0.58], a main effect of content domain (*F*_(1,29)_ = 4.56, *p* = 0.04, *η^2^_p_* = 0.14 [0.003–0.32]; all other effects *p* > 0.1) such that gender-related trials were associated with increased activity. To complement the findings, we conducted a whole-brain interaction analysis to examine whether any neural regions responded differently to the confirmation or violation of expectations between specific targets. Given the different experimental context between the two content domains, we first examined the interaction of expectations and specific targets within each content domain separately. This analysis yielded 3 regions (p<0.05, FWE-corrected; Table S5 and Figure S4), including the left inferior frontal gyrus (LIFG) and left superior temporal gyrus (LSTG), in which consistency with expectations yielded more activity than their violation only when the statements pertained to Trump (LIFG results: *F*_(1,114.24)_ = 40.12, *p* < 0.0001, *η^2^_p_* = 0.26 [0.15–0.36]) and not to any of the other targets (triple interaction: *F*_(1,29)_ = 33.32, *p* < 0.0001, *η^2^_p_* = 0.53 [0.30–0.66; similar patterns were observed in LSTG]. Disambiguating this triple interaction, we verified the interaction between expectation results and specific targets for leaders: *F*_(1,29)_ = 42.73, *p* < 0.0001, *η^2^_p_* = 0.6 [0.37–0.71]; the parallel interaction within gender content was not significant: *F*_(1,29)_ = 0.55, *p* > 0.4, *η^2^_p_* = 0.02 [0–0.03]). Notably, no main effect of expectation results was observed in these regions (all *p’s* > 0.05). Together, these findings suggest that the consistency with expectations pertaining to Donald Trump evokes an additional process which is not triggered for confirmation of other types of expectations.

*Behavioral analysis: Online studies*

In addition to the analyses reported in the main manuscript (for raw distribution data, see Table S6), in Study 4, we also performed an exploratory pre-registered analysis to examine the relationship between PSEs and support for the specific leader. To that end, we collected from each participant how much they liked Barack Obama and how much they liked Donald Trump on two separate 0–100 scales. Unsurprisingly for online data collection, our participants demonstrated a skewed preference toward Barack Obama (mean Obama liking for participants in Study 4a: 71.89, *S.D*. = 28.68; mean Trump liking for participants in Study 4b: 17.1, *S.D.* = 27.33). We correlated these data with PSEs for the corresponding leader in each study. As shown in Figure S3, the data are highly skewed, with many participants indicating the maximum liking rating for Obama and the minimum liking rating for Trump. As such, we do not provide inferential statistics as these data violate the statistical assumptions. Descriptively, we can see a small positive slope between PSE and liking for each leader. These descriptive trends can be taken to cautiously hint that the more one likes a leader, the more they would be willing to forgo money to see information consistent with the expectation from that leader.

**Supplementary Materials References**

Bainbridge, W.A., Isola, P., Oliva, A. (2013). The intrinsic memorability of face photographs. *Journal of Experimental Psychology: General*, **142**, 1323–34

Bartra, O., McGuire, J.T., Kable, J.W. (2013). The valuation system: A coordinate-based meta-analysis of BOLD fMRI experiments examining neural correlates of subjective value. *NeuroImage*, **76**, 412–27

Bates, D.M., Maechler, M., Bolker, B., et al. (2014). lme4: Linear mixed-effects models using Eigen and S4

Christensen, R.H.B. (2018). ordinal---Regression Models for Ordinal Data

Cusack, R., Papadakis, N. (2002). New robust 3-D phase unwrapping algorithms: Application to magnetic field mapping and undistorting echoplanar images. *NeuroImage*, **16**, 754–64

Dale, A.M. (1999). Optimal experimental design for event-related fMRI. *Human Brain Mapping*, **8**, 109–14

Feinberg, D.A., Moeller, S., Smith, S.M., et al. (2010). Multiplexed echo planar imaging for sub-second whole brain FMRI and fast diffusion imaging. *PloS one*, **5**, e15710

Glick, P., Fiske, S.T. (1996). The Ambivalent Sexism Inventory: Differentiating hostile and benevolent sexism. *Journal of Personality and Social Psychology*, **70**, 491–512

Ho, A.K., Sidanius, J., Kteily, N., et al. (2015). The nature of social dominance orientation: Theorizing and measuring preferences for intergroup inequality using the new SDO₇ scale. *Journal of Personality and Social Psychology*, **109**, 1003–28

Klonis, S.C., Plant, E.A., Devine, P.G. (2005). Internal and external motivation to respond without sexism. *Personality and Social Psychology Bulletin*, **31**, 1237–49

Knutson, B., Westdorp, A., Kaiser, E., et al. (2000). FMRI visualization of brain activity during a monetary incentive delay task. *NeuroImage*, **12**, 20–27

Kruglanski, A.W., Webster, D.M., Klem, A. (1993). Motivated resistance and openness to persuasion in the presence or absence of prior information. *Journal of Personality and Social Psychology*, **65**, 861–76

Lo, S., Andrews, S. (2015). To transform or not to transform : Using Generalized Linear Mixed Models to analyse reaction time data. *Frontiers in Psychology*, **6**, 1–16

Moeller, S., Yacoub, E., Olman, C.A., et al. (2010). Multiband multislice GE-EPI at 7 tesla, with 16-fold acceleration using partial parallel imaging with application to high spatial and temporal whole-brain fMRI. *Magnetic Resonance in Medicine*, **63**, 1144–53

Mumford, J.A., Davis, T., Poldrack, R.A. (2014). The impact of study design on pattern estimation for single-trial multivariate pattern analysis. *NeuroImage*, **103**, 130–38

Peirce, J.W. (2007). PsychoPy-Psychophysics software in Python. *Journal of Neuroscience Methods*, **162**, 8–13

Prentice, D.A., Carranza, E. (2002). What women and men should be, shouldn’t be, are allowed to be, and don’t have to be: the contents of prescriptive gender stereotypes. *Psychology of Women Quarterly*, **26**, 269–81

Xu, J., Moeller, S., Auerbach, E.J., et al. (2013). Evaluation of slice accelerations using multiband echo planar imaging at 3 T. *Neuroimage*, **83**, 991–1001

**Supplementary Materials Figures**

**
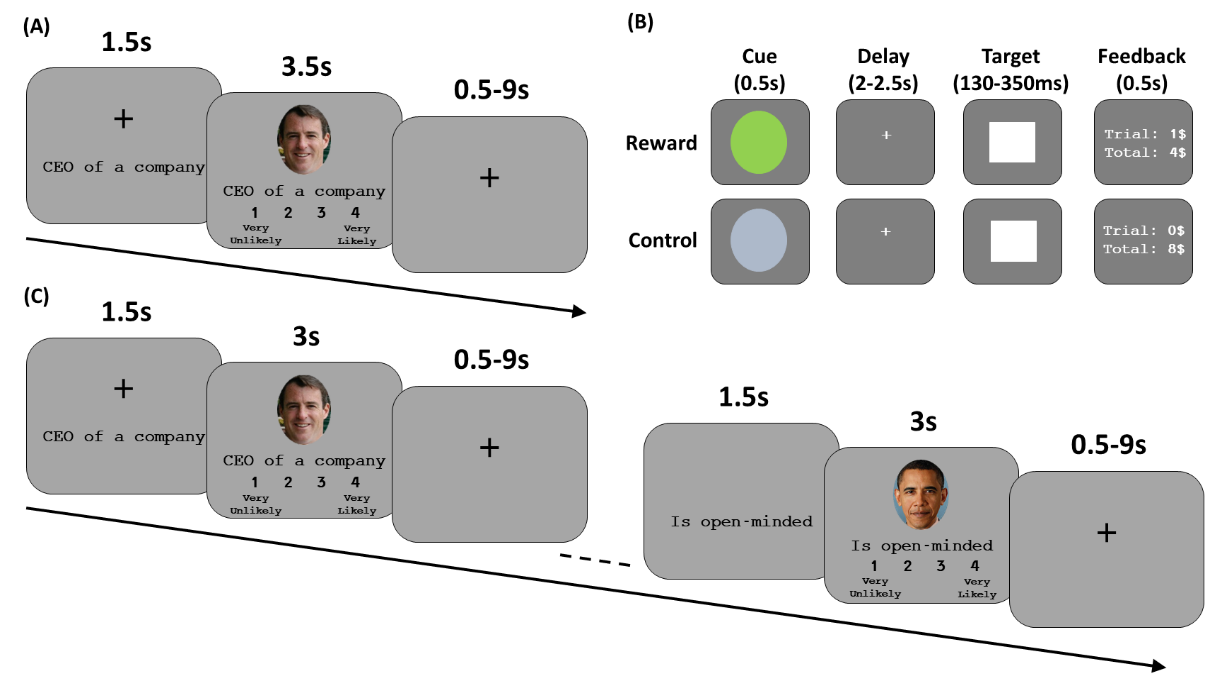

Fig. S1.** **Experimental designs of Studies 1 and 3.** (A) In Study 1, participants saw 204 unique trials. Each started with a gender-relevant or irrelevant statement, followed by a target face consistent with, violating, or neutral in respect to the displayed statement. Participants indicated how likely the presented person was to have the characteristic described in the statement on a 1 (“very unlikely”) to 4 (“very likely”) scale. (B) Following the impression formation task, participants completed the monetary incentive delay (MID) task. In each trial, participants saw a cue predicting the outcome of a successful response to the target. A green cue always indicated monetary reward, a blue cue always indicated no reward. Participants saw 30 reward cues and 15 no-reward cues. After a randomly jittered delay, a target appeared on screen for a brief duration (determined by a 2-up-1-down staircase procedure). Participants received feedback about their performance in each trial and across the entire task. (C) In Study 3, participants rated 240 trials, including 120 stereotype-related targets and 120 person-specific trials. Each content domain (stereotypes versus person-specific) was presented in separate blocks of 15 consecutive trials per type.

**
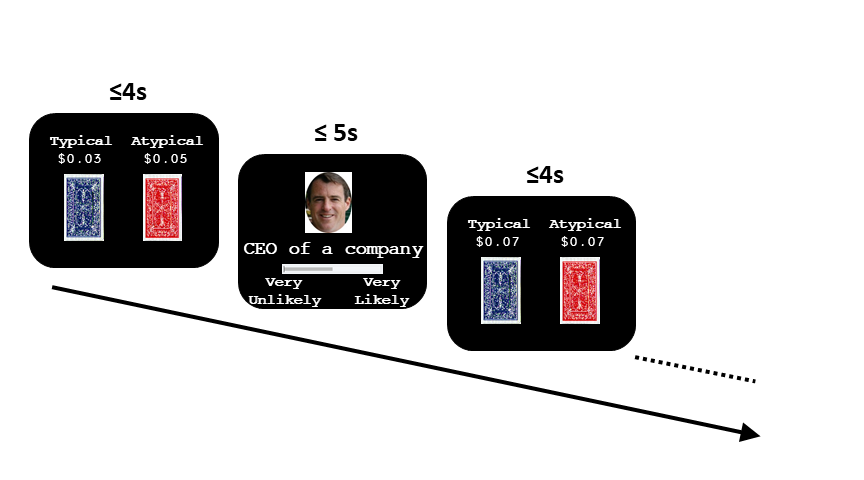
**

**Fig. S2.** **Experimental design of Study 2a**. Participants chose which target type to rate (typical, denoting stereotype-confirming targets, versus atypical, denoting stereotype-violating targets). Each target type was associated with a variable amount of money. After deciding which target type to rate, participants then rated a target of the chosen type. The design of studies 2b, 4a, and 4b followed this procedure.


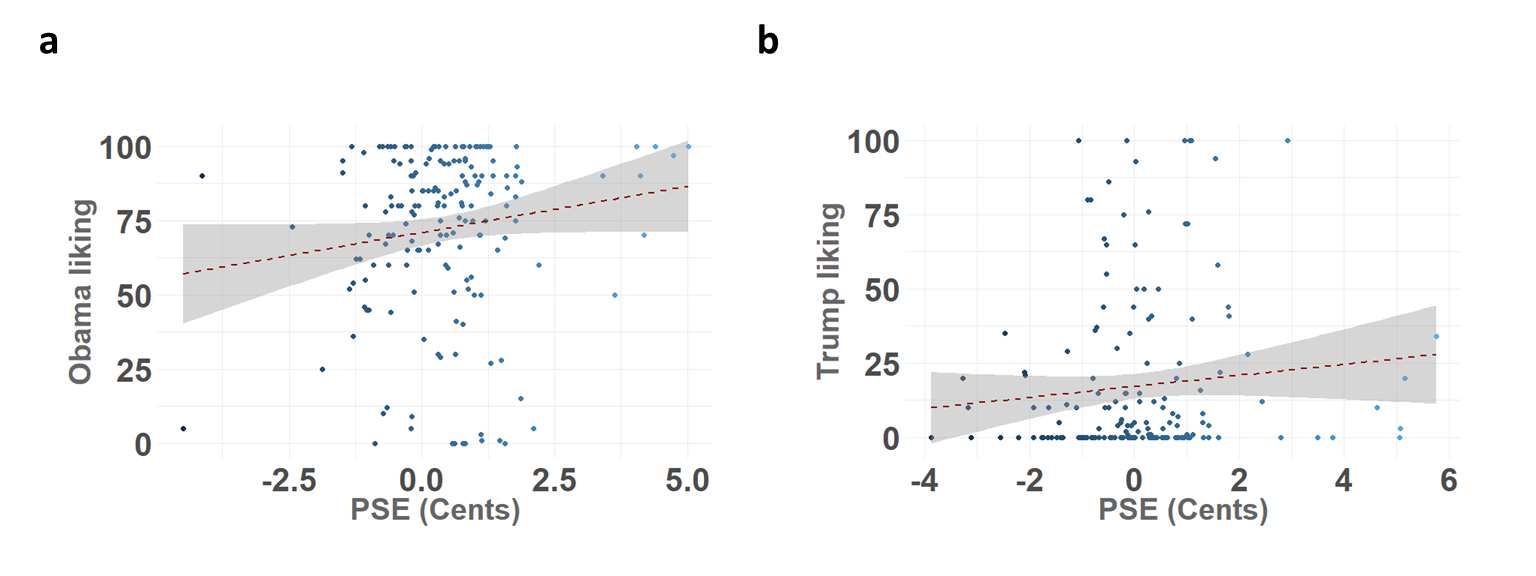


**Fig. S3.** **The correlations between the subjective value of expectation-consistent information and the liking of a specific leader in Study 4**. The subjective value was calculated as the point of subjective equivalence (PSE) per participant, as detailed in the main text. (a) Results from Study 4a, in which participants chose between seeing trials with expectation-consistent and expectation-inconsistent information about Barack Obama. (b) Results from Study 4b in which participants made equivalent choices about trials including Donald Trump. We do not provide inferential statistics as these data are heavily skewed.

**
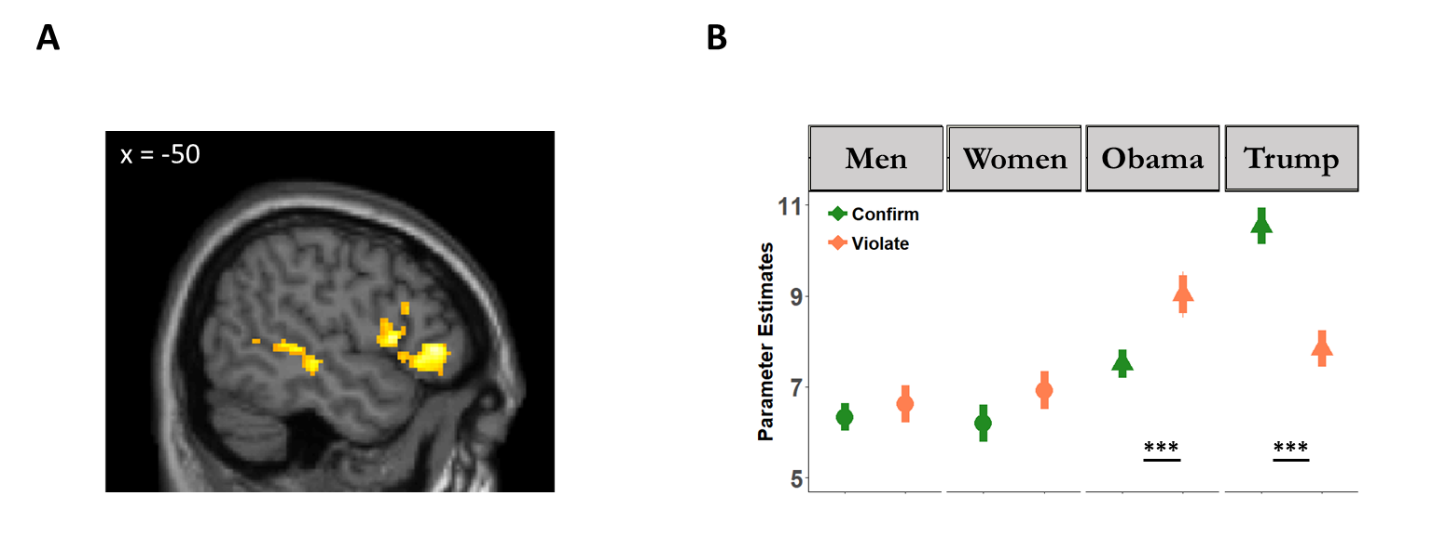
Fig. S4.** **Neural responses as a function of expectancy-outcome and type of target**. (A) results of a whole-brain analysis examining the interaction of outcome and type of target (see main text and Table S5 for details). (B) Parameter estimates drawn from the Left Inferior Frontal Gyrus ROI.

Table S1. Distribution of participants' ratings and reaction time (RT, in milliseconds) as a function of stereotypicality in the impression formation task in Study 1. Parentheses indicate standard error of the mean.
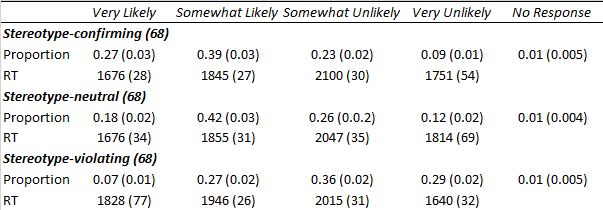


Table S2. Study 1: Gray matter regions showing differences in activity between stereotypical (stereotype-consistent) and counter-stereotypical (stereotype-violating) targets in model 1 (with no specification of behavioral response; p<0.05, corrected).

| ***Region*** | ***MNI coordinates*** | | | ***Z value*** | ***# voxels*** |
| --- | --- | --- | --- | --- | --- |
|  | ***X*** | ***Y*** | ***Z*** |  |  |
| *(a) Stereotypical > Counter-Stereotypical* | | | | | |
| Nucleus Accumbens | -2 | 4 | -6 | 4.98 | 30 |
| L Inferior Temporal | -50 | -56 | -12 | 4.03 | 108 |
|  |  |  |  |  |  |
| *(b) Counter-Stereotypical > Stereotypical* | | | | | |
| R Motor | 34 | -22 | 58 | 4.38 | 145 |
| R Inferior Frontal | 46 | 32 | -10 | 4.07 | 18 |
| Dorsomedial Prefrontal | 8 | 36 | 50 | 3.8 | 50 |
|  |  |  |  |  |  |
| *(c) Parametric Modulation (From Counter-Stereotypical to Stereotypical)* | | | | | |
| L inferior Parietal Sulcus | -30 | -82 | 36 | 4.22 | 17 |
| L Inferior Temporal | -48 | -58 | -12 | 4.19 | 60 |
| Extrastriate Cortex | 14 | -84 | -12 | 3.72 | 22 |
| Nucleus Accumbens | 0 | 6 | -6 | 4.15 | 20 |
| R Nucleus Accumbens | -8 | 8 | -4 | 3.91 | 13 |
| *(d) Parametric Modulation (From Stereotypical to Counter-Stereotypical)* | | | | | |
| R Motor | 34 | -20 | 58 | 4.41 | 128 |
| R Medial prefrontal | 12 | 48 | 12 | 4.33 | 18 |
| R Inferior Parietal | 58 | -50 | 38 | 3.94 | 18 |
| Dorsomedial Prefrontal | 8 | 36 | 50 | 3.85 | 16 |

Table S3. Distribution of participants' ratings and reaction time (RT, in milliseconds) as a function of expectation domain and expectation-consistency in the impression formation task in Study 3. Parentheses indicate standard error of the mean.


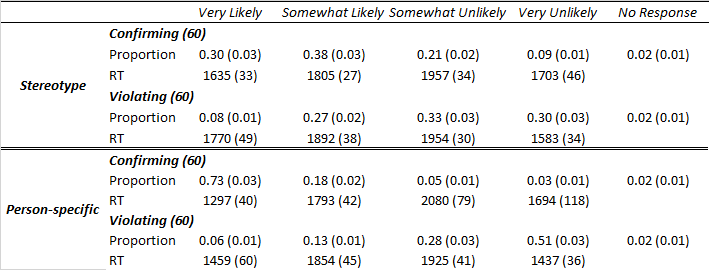


Table S4. Study 3: Gray matter regions showing differences in activity between expectation-consistent and expectation-violating targets. (a) and (b) portray the results collapsed across content domains, (c) through (f) include the results per content domain (p<0.05, corrected).

| ***Region*** | ***MNI coordinates*** | | | ***Z value*** | ***# voxels*** |
| --- | --- | --- | --- | --- | --- |
|  | ***X*** | ***Y*** | ***Z*** |  |  |
| *(a) Main effect: Confirming > Violating* | | | | | |
| Extrastriate Cortex | 12 | -80 | -4 | 5.63 | 2800 |
| R Motor | 46 | -14 | 52 | 5.43 | 298 |
| L Ventral Striatum | -12 | 20 | -2 | 4.93 | 89 |
| L Insula | -48 | 4 | 8 | 4.87 | 59 |
| Cerebellum | 10 | -74 | -38 | 4.82 | 56 |
| Cerebellum | -18 | -76 | -44 | 4.58 | 35 |
| L Occipitotemporal Cortex | -44 | -68 | 0 | 4.57 | 463 |
| R Ventral Striatum | 6 | 6 | -8 | 4.52 | 49 |
| R Insula | 36 | 6 | 10 | 4.51 | 40 |
| L Parietal Operculum | -44 | -32 | 20 | 4.34 | 166 |
| Occipital Cortex | 24 | -96 | 20 | 4.29 | 266 |
| Posterior Cingulate | -2 | -34 | 44 | 4.26 | 240 |
| R Fusiform Gyrus | 48 | -42 | -16 | 4.25 | 57 |
| R Occipitotemporal Cortex | 54 | -60 | 0 | 4.19 | 221 |
| L Superior Temporal | -66 | -36 | 20 | 4.08 | 62 |
| L Insula | -36 | -12 | 14 | 4.06 | 42 |
| L Parahippocampus | -24 | -40 | -6 | 4.06 | 50 |
| L Inferior Frontal | -42 | 38 | 12 | 4.01 | 34 |
| Precuneus | -16 | -62 | 16 | 3.98 | 103 |
| Ventromedial Prefrontal | 6 | 42 | -16 | 3.86 | 28 |
| L Motor | -50 | -16 | 54 | 3.77 | 112 |
| L Temporal Pole | -48 | 14 | -14 | 3.72 | 20 |
| R Orbitofrontal | 26 | 38 | -18 | 3.69 | 26 |
| R Inferior Frontal | 52 | 8 | -4 | 3.64 | 39 |
|  |  |  |  |  |  |
| *(b) Main effect: Violating > Confirming* | | | | | |
| Dorsomedial Prefrontal | 12 | 34 | 52 | 4.22 | 23 |
| R Motor | 36 | -24 | 54 | 3.99 | 50 |
| Dorsomedial Prefrontal | -8 | 42 | 50 | 3.92 | 62 |
| L Middle Frontal | -38 | 20 | 46 | 3.88 | 32 |
|  |  |  |  |  |  |
| *(c) Gender: Confirming > Violating* | | | | | |
| Extrastriate Cortex | 10 | -78 | -6 | 4.48 | 146 |
| R Occipitotemporal Cortex | 54 | -62 | 2 | 3.80 | 47 |
| Ventral Striatum | 4 | 10 | -10 | 3.78 | 21 |
| L Motor | 52 | -12 | 54 | 3.66 | 64 |
| R Lateral Occipital | 30 | -84 | 38 | 3.55 | 28 |
|  |  |  |  |  |  |
| *(d) Gender: Violating > Confirming* | | | | | |
| Dorsomedial Prefrontal | 12 | 34 | 52 | 3.92 | 33 |
| L Middle Frontal | -34 | 20 | 40 | 3.55 | 19 |
|  |  |  |  |  |  |
| *(e) Leaders: Confirming > Violating* | | | | | |
| Extrastriate Cortex | 8 | -76 | -6 | 5.56 | 1854 |
| R Motor | 48 | -14 | 52 | 5.42 | 204 |
| Posterior Cingualte | -8 | -40 | 54 | 4.34 | 57 |
| L Parietal Operculum | -42 | -36 | 26 | 4.31 | 248 |
| R Insula | 36 | 6 | 10 | 4.23 | 24 |
| L Temporal Pole | -50 | 12 | -12 | 4.19 | 80 |
| Cerebellum | 24 | -66 | -44 | 4.18 | 24 |
| R Supramarginal gyrus | 50 | -36 | 8 | 4.06 | 50 |
| L Inferior Frontal / Insula | -48 | 4 | 6 | 4.01 | 35 |
| L Occipitotemporal Cortex | -44 | -68 | 0 | 3.89 | 21 |
| Cuneus | -2 | -94 | 16 | 3.83 | 21 |
| R Fusiform Gyrus | 48 | -44 | -16 | 3.78 | 30 |
| Cerebellum | 8 | -74 | -38 | 3.76 | 20 |
| L Occipitotemporal Cortex | -48 | -58 | -2 | 3.71 | 111 |
| Precuneus | -14 | -66 | 28 | 3.71 | 55 |
| L Inferior Frontal | -60 | 6 | 10 | 3.68 | 22 |
| Precuneus | -14 | -78 | 42 | 3.62 | 74 |
| Ventral Striatum | -10 | 20 | -2 | 3.57 | 21 |
| Anterior Cingualte | -2 | 14 | 32 | 3.49 | 31 |
| R Inferior Frontal / Insula | 50 | 4 | -2 | 3.48 | 22 |
| L Motor | -50 | -16 | 54 | 3.45 | 22 |
| *(f) Leaders: Violating > Confirming* | | | | | |
| R Motor | 40 | -24 | 60 | 4.17 | 90 |
| Dorsomedial Prefrontal | -8 | 42 | 50 | 3.77 | 22 |

Table S5. Study 3: Results of a set of two whole-brain analyses examining the interactive effects of expectation-consistency and specific target, done separately for gender and leaders. (p<0.05, corrected).

| ***Region*** | ***MNI coordinates*** | | | ***Z value*** | ***# voxels*** |
| --- | --- | --- | --- | --- | --- |
|  | ***X*** | ***Y*** | ***Z*** |  |  |
| *(a) Gender: Confirmation effect in men > Confirmation effect in women* | | | | | |
| None |  |  |  |  |  |
|  |  |  |  |  |  |
| *(b) Gender: Confirmation effect in women > Confirmation effect in men* | | | | | |
| None |  |  |  |  |  |
|  |  |  |  |  |  |
| *(c) Leaders: Confirmation effect in Obama > Confirmation effect in Trump* | | | | | |
| None |  |  |  |  |  |
|  |  |  |  |  |  |
| *(d) Leaders: Confirmation effect in Trump > Confirmation effect in Obama* | | | | | |
| Cuneus | 12 | -88 | 24 | 5.16 | 435 |
| L Inferior Frontal | -50 | 34 | 2 | 4.83 | 473 |
| L Superior Temporal | -62 | -36 | 4 | 4.32 | 352 |
| Extrastriate Cortex | -6 | -80 | 6 | 3.67 | 75 |

*Table S6.* Distribution of proportions of trials in Studies 2 and 4 in which participants chose to see an expectation-confirming target. Data are presented as a function of the difference in monetary value between the two decks of cards.

| ***Monetary Difference*** | ***N*** | ***% Consistent Chosen*** | ***S.D.*** | ***CI*** |
| --- | --- | --- | --- | --- |
|  |  |  |  |  |
| **Study 2a** | | | | |
|  |  |  |  |  |
| -6 | 173 | 0.124 | 0.318 | 0.048 |
| -4 | 174 | 0.149 | 0.301 | 0.045 |
| -2 | 174 | 0.187 | 0.268 | 0.040 |
| 0 | 174 | 0.577 | 0.246 | 0.037 |
| 2 | 174 | 0.843 | 0.253 | 0.038 |
| 4 | 174 | 0.885 | 0.273 | 0.041 |
| 6 | 171 | 0.919 | 0.270 | 0.041 |
|  |  |  |  |  |
| **Study 2a** | | | | |
|  |  |  |  |  |
| -6 | 167 | 0.084 | 0.274 | 0.042 |
| -4 | 169 | 0.145 | 0.284 | 0.043 |
| -2 | 169 | 0.198 | 0.238 | 0.036 |
| 0 | 169 | 0.573 | 0.273 | 0.041 |
| 2 | 169 | 0.848 | 0.217 | 0.033 |
| 4 | 169 | 0.906 | 0.229 | 0.035 |
| 6 | 166 | 0.902 | 0.294 | 0.045 |
|  |  |  |  |  |
| **Study 4a** | | | | |
|  |  |  |  |  |
| -6 | 187 | 0.070 | 0.185 | 0.027 |
| -4 | 189 | 0.110 | 0.177 | 0.025 |
| -2 | 189 | 0.148 | 0.204 | 0.029 |
| 0 | 189 | 0.603 | 0.253 | 0.036 |
| 2 | 189 | 0.895 | 0.172 | 0.025 |
| 4 | 189 | 0.910 | 0.182 | 0.026 |
| 6 | 189 | 0.938 | 0.179 | 0.026 |
|  |  |  |  |  |
| **Study 4b** | | | | |
|  |  |  |  |  |
| -6 | 172 | 0.089 | 0.196 | 0.030 |
| -4 | 172 | 0.107 | 0.190 | 0.029 |
| -2 | 172 | 0.156 | 0.192 | 0.029 |
| 0 | 172 | 0.491 | 0.247 | 0.037 |
| 2 | 172 | 0.876 | 0.179 | 0.027 |
| 4 | 172 | 0.911 | 0.163 | 0.024 |
| 6 | 172 | 0.904 | 0.226 | 0.034 |
